# Identification and Characterization of Cyprinid Herpesvirus-3 (CyHV-3) Encoded MicroRNAs

**DOI:** 10.1371/journal.pone.0125434

**Published:** 2015-04-30

**Authors:** Owen H. Donohoe, Kathy Henshilwood, Keith Way, Roya Hakimjavadi, David M. Stone, Dermot Walls

**Affiliations:** 1 Marine Institute, Rinville, Oranmore, Co. Galway, Ireland; 2 School of Biotechnology and National Centre for Sensor Research, Dublin City University, Dublin, Ireland; 3 Centre for Environment, Fisheries and Aquaculture Science (Cefas), The Nothe, Weymouth, Dorset, the United Kingdom; French National Center for Scientific Research - Institut de biologie moléculaire et cellulaire, FRANCE

## Abstract

MicroRNAs (miRNAs) are a class of small non-coding RNAs involved in post-transcriptional gene regulation. Some viruses encode their own miRNAs and these are increasingly being recognized as important modulators of viral and host gene expression. Cyprinid herpesvirus 3 (CyHV-3) is a highly pathogenic agent that causes acute mass mortalities in carp (*Cyprinus carpio carpio*) and koi (*Cyprinus carpio koi*) worldwide. Here, bioinformatic analyses of the CyHV-3 genome suggested the presence of non-conserved precursor miRNA (pre-miRNA) genes. Deep sequencing of small RNA fractions prepared from *in vitro* CyHV-3 infections led to the identification of potential miRNAs and miRNA–offset RNAs (moRNAs) derived from some bioinformatically predicted pre-miRNAs. DNA microarray hybridization analysis, Northern blotting and stem-loop RT-qPCR were then used to definitively confirm that CyHV-3 expresses two pre-miRNAs during infection *in vitro*. The evidence also suggested the presence of an additional four high-probability and two putative viral pre-miRNAs. MiRNAs from the two confirmed pre-miRNAs were also detected in gill tissue from CyHV-3-infected carp. We also present evidence that one confirmed miRNA can regulate the expression of a putative CyHV-3-encoded dUTPase. Candidate homologues of some CyHV-3 pre-miRNAs were identified in CyHV-1 and CyHV-2. This is the first report of miRNA and moRNA genes encoded by members of the *Alloherpesviridae* family, a group distantly related to the *Herpesviridae* family. The discovery of these novel CyHV-3 genes may help further our understanding of the biology of this economically important virus and their encoded miRNAs may have potential as biomarkers for the diagnosis of latent CyHV-3.

## Introduction

MicroRNAs (miRNAs) are a class of small non-coding RNAs involved in the regulation of gene expression in animals and plants [1–4]. They are ~22 nucleotides (nt) in length and are typically encoded within intronic or intergenic regions of genomes [5–7]. MiRNAs are derived from longer primary miRNA transcripts (pri-miRNAs) which are processed by the nuclear RNase III enzyme, Drosha, to release precursor miRNA (pre-miRNA) hairpins (~60–70 nt) [8,9]. These are transported from the nucleus to the cytoplasm where they are cleaved near the terminal loop by another RNase III enzyme, Dicer, thus releasing a mature double-stranded miRNA duplex [10–12]. One of these strands is subsequently incorporated into a multi-protein complex known as the RNA-Induced Silencing Complex (RISC) [13–15]. Once incorporated into the RISC, miRNAs base pair with specific target messenger RNAs (mRNAs), resulting in gene specific silencing either through enhanced transcript degradation or translational inhibition, mediated by RISC components [16–18]. Each miRNA is usually accompanied by several less abundant ‘isomiRs’ that display differing degrees of terminal heterogeneity [19]. In addition to miRNAs, other small non-coding RNAs known as miRNA-offset-RNAs (moRNAs) can also be processed from some pre-miRNAs [20–22].

In recent years, some viruses have also been found to encode miRNAs [23]. These have been shown to regulate both host and viral gene expression leading to enhanced immune evasion, the manipulation of host cell fate and regulation of the viral lytic cycle [24–27]. MiRNA genes are far more prevalent among members of the *Herpesviridae* family [24–28]. This may partly be a result of their distinctive biological characteristics, including large double-stranded DNA (dsDNA) genomes, nuclear replication, and their hallmark capacity to establish long-term latent infections, during which there is a need to maintain a cellular homeostasis that facilitates latency without using (or limiting the use of) potentially immunogenic viral proteins.

The *Alloherpesviridae* family includes viruses that infect lower vertebrates such as fish and amphibians. Most known members have been identified in economically important fish species [29]. The *Alloherpesviridae* are very distantly related to the *Herpesviridae* with convincing evidence for only one gene being conserved between the two families [30–32]. Remarkably, despite the high degree of evolutionary divergence, both families share many of the same biological characteristics [29,30], however there are no reports to date regarding the miRNA-coding capacity of the *Alloherpesviridae*. Cyprinid Herpesvirus-3 (CyHV-3) is a highly pathogenic member of the *Alloherpesviridae* that causes acute mass mortalities in populations of common carp (*Cyprinus carpio carpio*) and koi (*Cyprinus carpio koi*), a condition referred to as Koi Herpesvirus Disease or KHVD. It has also been shown to asymptomatically infect other fish species (for reviews, see [33–37]). During a typical outbreak, mortality rates among affected populations are over 80% [38–41], thus CyHV-3 is recognized as a significant problem for the common carp and ornamental koi aquaculture industries [42], recently estimated to be worth over US$4.5 billion and US$15 billion, respectively. As with other farmed fish species, threats to the long-term feasibility of carp aquaculture are becoming ever more relevant, especially in the context of global food security [43]. Like all other herpesviruses, CyHV-3 appears to establish long-term latent infections. Outside the permissive water temperature range of 16–28°C, mortalities decrease but low levels of CyHV-3 DNA can often still be detected in healthy survivors long after initial exposure to the virus [44,45] particularly in white blood cells [46,47], suggesting they survive as latently-infected carriers. Furthermore, CyHV-3 lytic infections can be reactivated in such carriers through re-exposure to permissive temperatures [44,48] thus clearly showing that such fish can act as reservoirs of infectious virus during non-permissive conditions.

Here, we use a range of bioinformatic and molecular methods to show that CyHV-3 encodes its own miRNAs, associated isomiRs and moRNAs, and that they are expressed *in vitro* and *in vivo*. We present evidence to show that the most highly expressed CyHV-3 miRNA can downregulate the viral dUTPase-coding gene, *ORF123*, through interaction with a target site in the *ORF123* 3′ UTR. Pre-miRNA homologues were also identified in the two closely related viruses *Cyprinid Herpesvirus 1* (CyHV-1) and *Cyprinid Herpesvirus 2* (CyHV-2), indicating that other members of the *Alloherpesviridae* may also encode miRNAs. This is the first report of miRNAs being produced by a member of the *Alloherpesviridae* family.

## Materials and Methods

### *De novo* pre-miRNA prediction

Putative pre-miRNA-coding regions on viral genomes were predicted using VMir Analyser (version 2.3) [49]. The specific prediction parameters used were as follows: maximum size for calculation of score—100; maximum size for assigning assign subsidiary hairpins—50; maximum size for assigning repeat hairpins—50; minimum hairpin size—any; maximum hairpin size—any; minimum hairpin score—any.

### Cell culture, virus infections and fish

The common carp brain (CCB) cell line [50] was cultured at 20°C in 75 cm^2^ cell culture flasks using Eagle’s Minimum Essential Medium with Earle’s salts, supplemented with 25 nM NaHCO_3_, 25 mM HEPES, 10% v/v Foetal Bovine Serum, 2 mM GlutaMAX Supplement, 1 x Non-essential amino acids and 250 units/ mL Penicillin/250 μg/ mL Streptomycin (Pen/Strep). Human Embryonic Kidney (HEK293) cells were grown in Dulbecco’s Modified Eagle medium (DMEM) supplemented with 10% FBS and Pen/Strep as above. CCBs were used for the culture of two separate CyHV-3 isolates, namely H361 and N076 (which were KHV-U/I and KHV-U/I/TUMST1 strain intermediates, respectively). To establish non-synchronous lytic infections, flasks freshly seeded with CCB cells were incubated for 24 hours (h) at 27°C (~70% confluent or 5.7–6.9 x10^6^ cells) and inoculated with 1 mL preparations of either H361 (2.7 x 10^4^ TCID_50_/mL) or N076 (5 x 10^5^ TCID_50_/mL) isolates and maintained at 22°C, thus allowing the virus to enter the lytic cycle.

All carp tissue samples used had been previously submitted to the Cefas laboratory (Weymouth, UK) for diagnostic investigation purposes. Fish that displayed clinical symptoms typical of KHVD were obtained from sites of suspected KHVD outbreaks in the UK during 2014. Gill tissue samples from uninfected common carp were obtained from a coarse fish farm in Southern England (see Dataset T in S1 File for more details relating to the origin of all carp samples). The facilities at Cefas where the uninfected fish were euthanized holds an establishment license under the Animal Scientific Procedures Act (ASPA) and are therefore licensed to carry out schedule 1 euthanasia. Carp were sampled following terminal anaesthesia with ethyl 4-aminobenzoate (Benzocaine, 150 mg/L) and destruction of the brain. Samples were screened for CyHV-3 using susceptible cell lines. Briefly, gill tissue samples (not exceeding 1g in total) from individual fish were placed in 9 mL of viral transport medium consisting of Glasgow modification of minimal essential medium (GMEM) supplemented with 10% new-born calf serum, 2 mM L-glutamine and 1% antibiotic + antimycotic solution (all from Sigma) at a temperature not exceeding 8°C. Tissues were homogenised with a pestle, mortar and sterile sand and re-suspended in the original transport medium. Gill homogenates were clarified by centrifugation at 2000 x g for 20 minutes (min), clarified supernatants were filtered through a 0.45 mm disposable cellulose acetate filter unit (Minisart, Sartorius) and 200 μL aliquots of filtrate were used to inoculate koi fin (cultured as per [39]) and CCB cell monolayers in 25 cm^2^ tissue culture flasks (Falcon). All tissue homogenates, including those prepared in the same way from uninfected control fish, were screened for CyHV-1, CyHV-2 and CyHV-3 using a PCR targeting the DNA polymerase gene in all 3 viruses [51], followed by sequencing of PCR products. This confirmed the presence of CyHV-3 in samples from KHVD fish, its absence in negative control fish and the absence of CyHV-1 and CyHV-2 in all samples.

### Nucleic acid isolation and size fractionation

DNA was extracted from all samples using the DNeasy Tissue Kit (Qiagen) as per manufacturer’s instructions. RNA was extracted from CCB cells (infected/non-infected) using the miRNeasy Mini Kit (Qiagen) following the manufacturer’s protocol. Total RNA was prepared from carp gill tissue homogenates by adding 250 μL of homogenate to 1 mL Trizol (Life Technologies) followed by extraction as per manufacturer’s protocol. Total RNA samples (or ligation products; see cDNA library preparation below) were fractionated by polyacrylamide gel electrophoresis (PAGE; 15% Polyacrylamide/7M Urea). Molecular weight estimations were made with the aid of pre-stained RNA size markers (Small RNA Plus, Biodynamics Laboratory Inc.) and gel sections containing short RNAs (or ligation products) were excised accordingly. RNA was extracted from gel slices as described elsewhere [52] and eluted in 100 μL of 10 mM Tris-HCl pH 7.5. Extracted RNA was concentrated as required by ethanol (EtOH) precipitation by adjusting to 0.3 M NaOAc, supplemented with 5 μg/ mL linear acrylamide (Ambion) followed by addition of MBG EtOH to 75% v/v and incubation at -80°C for ≥2 h. RNA pellets were then collected by centrifugation, washed with 80% MBG EtOH, air dried and resuspended in 10 mM Tris-HCl pH 7.5.

### cDNA library preparation and small RNA deep sequencing

Two separate CyHV-3 *in vitro* lytic infections were prepared using the H361 and N076 isolates and total RNA was extracted at 18 and 14 days post-infection (dpi), respectively, followed by RNA size fractionation and isolation of 17–25 nt RNA. Two small RNA deep sequencing libraries were prepared from these miRNA-enriched samples, using an adaptation of the Illumina Small RNA v1.5 protocol (see Dataset A in S1 File for adaptors and primers used). There were four stages to this (i) 3′ Adaptor ligation: For each sample, 70–200 ng of 17–25 nt RNA was mixed with 10 pMol of 3′ adaptor in a total volume of 10 μL, the mixture was heated to 70°C for 2 min and placed on ice for 1 min. This was then added to 10 μL 3′ end ligation reaction mix consisting of 20 U/ μL T4 Ligase 2 (NEB), 2x T4 Ligase 2 Buffer, 8mM MgCl_2_, 20% v/v DMSO, 14.5% v/v PEG 8000 (Sigma) and 4 U/ μL RNasin (Promega) (giving a total reaction volume of 20 μL), followed by incubation of the ligation reaction at 22°C for 2 h. The resulting 3′ adaptor ligation products (~34–42 nt) were PAGE purified and resuspended in 8 μL of 10 mM Tris-HCl. (ii) 5′ Adaptor ligation: The 3′ adaptor ligation products were then mixed with 10 pMol of 5′ adaptor in a total volume of 10 μL, the mixture was heated to 70°C for 2 min and placed on ice for a further 1 min. This was then added to 10 μL 5′ end ligation reaction mix consisting of 2 U/ μL T4 Ligase 1 (NEB), 2x T4 Ligase I Buffer, 20% v/v DMSO, 22.5% v/v PEG 8000 (Sigma), 4 U/ μL RNasin (Promega) (giving a total reaction volume of 20 μL), followed by incubation at 37°C for 1 h. The resulting 5′ adaptor ligation products (~60–68 nt) were isolated and resuspended as described for the 3′ ligation products above. (iii) Reverse transcription (RT): The 5′/3′ adaptor ligation products were used as templates for RT. To this end, these were mixed with 10 pMol of Library prep. RT Primer in a total volume of 10 μL and the mixture was heated to 70°C for 2 min and placed on ice for a further 1 min. This was added to 10 μL RT mix consisting of 2 mM dNTPs, 20 U/ μL SuperScript III RT (Invitrogen), 2x SS III RT Buffer, 0.10 M DTT (Promega), 4 U/ μL RNasin (Promega) (giving a total reaction volume of 20 μL). The RT reaction was carried out using the following thermocycler programme: 55°C for 90 min and 70°C for 15 min. (iv) PCR: 20 μL of RT product was used as input for PCR. This was made up to 25 μL with H_2_O and added to 25 μL RT mix consisting of 2X Phusion HF Buffer, 0.4 μM each of Forward Primer and Reverse Primer, 500 uM dNTPs 0.04 U/ μL Phusion DNA Polymerase (Finnzymes) (giving a total reaction volume of 50 μL). Thermocycling conditions were: 98°C for 30 seconds (s) followed by 12 cycles of 98°C for 10 s, 60°C for 30 s, 72°C for 15 s and a final elongation step of 72°C for 10 min. The resulting dsDNA libraries were sequenced using an Illumina Genome Analyser in the TrinSeq core facility (Trinity College Institute for Molecular Medicine, St James’s Hospital, Dublin, Ireland).

### Analysis of small RNA deep sequencing data and pre-miRNA identification

Fastaq files were collapsed into sequence tag files and intact adaptor sequences were trimmed from the 3′ ends. Pre-miRNA candidates were then identified using two different methods. Firstly, a ‘non-automated approach’ was taken as follows: collapsed sequence tags were given unique IDs (incorporating read count information), converted into FASTA format using tabtofasta script (tabtofasta.pl http://nebc.nerc.ac.uk/tools/code-corner/scripts/sequence-formatting-and-other-text-manipulation) and mapped to the CyHV-3 genome (GeneBank Accession No. DQ657948.1) and CyHV-3 open reading frame (ORF) sequences separately using SeqMap [53] (version 1.0.12). Mapped transcript reads were arranged in an Excel file according to DNA strand and genome position and details of any ORF overlap were noted. The same mapped data was then un-collapsed based on read count information using the FASTX uncollapser script (FASTX-Toolkit version 0.0.13 [http://hannonlab.cshl.edu/fastx_toolkit/index.html]) and visualised using SeqMonk (version 0.16.0 [http://www.bioinformatics.babraham.ac.uk/projects/seqmonk]). ‘Excel-arranged’ and ‘SeqMonk-visualised’ mapped data were used in unison to identify loci that were specifically enriched for small RNA reads. Within such loci, viral transcripts showing no ORF overlap and read counts ≥10 were selected for cross-referencing with VMir-predicted pre-miRNA loci. High-probability pre-miRNA candidates were then identified based on compliance with all of the following nine criteria: (i) presence of small RNAs (i.e. putative miRNAs) mapping to the upper stem of the pre-miRNA candidate hairpin structure (either 5′ or 3′ arm) adjacent to the terminal loop (additional transcripts mapping to the stem but adjacent to putative miRNAs were recorded as moRNAs); (ii) at least one of the putative miRNAs mapping to a given pre-miRNA candidate had a read-count of ≥10 in at least one infection experiment; (iii) the extended pre-miRNA candidate sequence did not overlap with any known CyHV-3 ORFs; (iv) each putative miRNA was accompanied by miRNA/miRNA* reads from the opposite arm of the pre-miRNA in both infection experiments; (v) on each pre-miRNA arm, the same read (or its isomiR, differing by a maximum of +/- 1 nt at the 5′ end) was identified as the putative miRNA (the dominant read at that locus) in both infection experiments; (vi) the pre-miRNA candidate itself was classified as a real pre-miRNA by both MiPred [54] and CSHMM [55]; (vii) the pre-miRNA candidate structure had a minimum free energy (MFE) < -25 kcal/mol that was statistically significant, as determined by MiPred; (viii) the alignment signatures of putative miRNAs/moRNAs and isomiRs/isomoRs to pre-miRNA candidates was consistent with the currently accepted model of miRNA biogenesis outlined elsewhere [56] and (ix) reads mapping to pre-miRNA candidate loci collectively formed discrete stacks and discrete regions of read enrichment relative to flanking genomic loci. A second ‘automated approach’ was also taken to identify pre-miRNA candidates: the same collapsed sequence tag and mapped data was reformatted appropriately for use as input for the miRDeep2 pipeline (version 0.0.5) [57,58] and MIREAP (version 0.2) [59]. High-probability pre-miRNA candidates were only described as such if either identified independently by both programmes or multiple times by the same program. Further to the above ‘automated’ and ‘non automated’ approaches, the complete set of isomiRs associated with each high-probability CyHV-3 miRNA were analysed in terms of 5′ and 3′ end heterogeneity (with isomiR sets from different infection experiments analysed separately). Individual isomiR sets were compared using three different ratios, Ratio-1 (ratio of the number of unique isomiRs offset at the 3′ end to those offset at the 5′ end), Ratio-2 (ratio of the average 3′ offset for each isomiR to the average 5′ offset) and Ratio-3 (ratio of combined read count of all isomiRs offset at the 3′ end to that of all isomiRs offset at the 5′ end)

### DNA Microarray analysis

DNA oligonucleotide probes were designed using the VMir probe design tool. Each probe was complementary to a region spanning 30 nt of the corresponding stem starting from the loop apex (i.e. complementary to putative miRNAs). Probes targeting low and high probability CyHV-3 pre-miRNA candidates identified using the ‘non-automated’ approach and the 2,893 highest-scoring VMir predicted CyHV-3 pre-miRNAs were included on the array. This strategy facilitated the probing of (i) highly abundant small RNAs (read count >10) mapping to either high or low-probability pre-miRNAs (ii) small RNAs that also mapped to either high or low-probability pre-miRNAs but were disregarded due to low abundance and (iii) theoretical miRNAs from other pre-miRNA predictions that were not represented in the sequencing data. The VMir probe design tool was also used to design associated scrambled and mismatched (by 1 nt every 5th nt) control probes for all miRNA probes. Each pre-miRNA also had associated flanking controls (targeting sequences 25 nt upstream and 25 nt downstream of the 5′ and 3′ miRNA probe target sequences respectively). Probes were also designed to target an exogenous spike-in control (a synthetic RNA oligonucleotide based on *Arabidopsis thaliana* miRNA ath-miR15.6g, miRBase accession MIMAT0001012) and several endogenous positive controls (host miRNA sequences ccr-miR-21, ccr-miR-22a, ccr-let-7a and ccr-et-7b; miRBase accessions MIMAT0026267, MIMAT0026275, MIMAT0026189 and MIMAT0026190 respectively). Custom oligonucleotide arrays (30K format) were synthesised by MYcroarray (Ann Arbor, MI) with array positions customised for use with a Tecan HS 400 Pro Hybridization Station. Each probe was also synthesized with a 30 nt spacer (Poly Ts) between the 3′ end and the array surface in order to eliminate the potential for reduced hybridization accessibility at extreme 3′ ends [60]. Two separate RNA size fractions, F1 (17–25 nt) and F2 (26–35 nt), were isolated from total RNA derived from CyHV-3 infected CCB cells, in this case the total RNA used was from the same H361 sample that was also used for small RNA deep sequencing. An additional RNA size fraction, F3 (17–25 nt), was isolated from non-infected CCB cells. Approximately 400 ng RNA from each sample (along with 200 aMol of the exogenous control) was non-enzymatically labelled and purified using the Label IT miRNA Labeling Kit Version 2 (Mirus Bio) according to the standard manufacturer’s protocol with modifications: 5x final concentration of Label IT Reagent was used so as to achieve a maximal labelling density and labelled RNA was eluted in 45 μL Elution buffer following purification. Forty microlitres of labelled RNA was added to 100 μL of hybridization buffer (final concentration of 2.5x SSPE, 20% Formamide, 0.01% Tween-20, 0.01 mg/mL Acetylated BSA, 6.5% w/v Dextran Sulphate and 2% SDS), this was then heated to 65°C for 5 min, kept on ice for 5 min and hybridized to the array at 42°C for 8 h in a Tecan HS 400 Pro Hybridization Station. Arrays were subsequently washed therein with 2x SSPE, 0.1% SDS at 30°C for 5 min, 1x SSPE at 22°C for 5 min and finally with 0.1x SSPE at 22°C for 3 min followed by Nitrogen drying. Arrays were scanned using a NovaRay Scanner (Alpha Innotech) with 8 s exposures at 5 μm resolution. Raw fluorescence data was imported into VMir and background values were established for each hybridization based on the median signals obtained from control probes. VMir was also used to process signals from probes targeting miRNAs and to correct them for potential non-specific hybridization where applicable using signals from corresponding control probes. Cut-off values for corrected miRNA probe signals [expressed as multiples of background (MOB)] were established for each hybridization based on the intrinsic variability of the signals from the control probes. This was done independently of VMir by calculating their average absolute deviation from the median value (background) and setting the cut-off values for corrected miRNA probe signals as one absolute deviation above the background in each hybridization. Only corrected miRNA probe signals that were above these cut-off values were scored as positive. MiRNA probes displaying positive signals using labelled RNA from F1 were provisionally attributed to CyHV-3 miRNAs if the same probes showed no corresponding positive signals with the F2 and F3 samples.

### Northern Blotting

Ten microgram samples of total RNA were fractionated by PAGE as described above, transferred to a BrightStar-Plus Positively Charged Nylon Membrane (Ambion) by electroblotting and crosslinked in an oven at 80°C for 4 h. Probes consisted of custom 5′ biotin labelled DNA oligos (IDT) complementary to high probability CyHV-3 miRNAs MR5057-miR-3p, MD11776-miR-3p, MD1111-miR-3p and MR5075-miR-3p (based on sequences from the H361 infection) and the host miRNA ccr-let-7a-5p. Lyophilised probes were resuspended at 1 nmol/μL. Membranes were pre-hybridized in 9 mL ULTRAhyb-Oligo buffer (Ambion) at 37°C for 30 min with gentle rocking. Prior to hybridization, 1 μL of each probe stock was diluted in 1 mL of pre-hybridization buffer, added to the blot and incubated at 37°C for 8–12 h with gentle rocking. This was followed by two 5 min washes with 20 mL 2X SSPE/0.1% SDS at room temperature and one 2 min wash with the same buffer at 37°C. Hybridized biotin labelled probes were detected using the BrightStar BioDetect Kit (Ambion) according to manufacturer’s protocol. Chemiluminescent signals were visualised using a BioLuminizer (CAMAG).

### Stem-loop reverse-transcription real-time PCR (RT-qPCR) and qPCR assays

Custom stem-loop RT primers and corresponding PCR primers/probes were designed using the Custom TaqMan Small RNA Assay Design Tool (Life Technologies). The CyHV-3 high probability miRNA sequences targeted were based on those identified in the H361 infection. The *C*. *carpio* miRNA ccr-let-7a-5p was detected using a hsa-let-7a-5p TaqMan MicroRNA Assay (Life Technologies, Assay ID 000377). RT and PCR steps for all TaqMan Small RNA Assays were done using MultiScribe RT and TaqMan Universal PCR Master mixes, respectively, according to the manufacturer’s protocol (TaqMan Small RNA Assay Protocol, Life Technologies, Rev. E01/2011). PCR was done using an Applied Biosystems 7500 instrument and associated software (version 2.0.1). The resulting normalised raw florescence data (Rn values) was imported into LinReg-PCR [61] for calculation of PCR efficiency and subsequent calculation of expression levels. Only samples showing signals in all replicates were considered valid. Where appropriate, CyHV-3 miRNA expression levels were normalised to levels of ccr-let-7a-5p in the same samples. QPCR assays used to target both CyHV-3 and carp genomic DNA were carried out as described elsewhere [45]. Viral DNA levels were expressed as linear scale values (using the formula 2^-Ct^) and normalised to corresponding levels of carp genomic DNA in the same samples, allowing comparison of relative levels between samples.

### Identification of pre-miRNA/miRNA homologues and miRNA target sites

CyHV-3 pre-miRNA sequences were aligned to the CyHV-1 (GeneBank Accession No. JQ815364.1) and CyHV-2 (GeneBank Accession No. JQ815363.1) genomes using the Basic Local Alignment Search Tool (BLAST) [62]. CyHV-3 pre-miRNA input sequences did not extend beyond the boundaries of the miRNAs (as only these regions are likely to be conserved [63]), except where moRNAs were present on the same pre-miRNA. All *C*. *carpio* and viral miRNAs were retrieved from miRBase (Release 19) in FASTA format, converted to tab delimited format using the fastatotab script (fastatotab.pl http://nebc.nerc.ac.uk/tools/code-corner/scripts/sequence-formatting-and-other-text-manipulation), and seeds (positions 2–7) were isolated using Excel by inserting column breaks between characters 1–2 and 8–9. Identifiers and seeds were converted back to FASTA using the tabtofasta script and aligned to CyHV-3 miRNA seeds using SeqMap (max. 1 mismatch). CyHV-3 3′ untranslated regions (UTRs) downstream of each CyHV-3 ORF were individually retrieved following importation of the CyHV-3 genome (GeneBank Acession No. DQ657948.1) into SnapGene Viewer (v2.3). Candidate CyHV-3 mRNA targets were identified using the TargetScan pipeline (version 6.0) [64–66] and PITA (version 6) [67].

### Vectors, western blots and luciferase reporter assays

The vector pCyHV-3-rdUTPase was designed to encode a recombinant N-terminally epitope-tagged CyHV-3 deoxyuridine triphosphatase (rdUTPase) expressed from *ORF123* and linked to its own 3′ UTR. To this end a DNA sequence was designed with the following layout: Kozak sequence/Hexahistidine tag/spacer/MYC tag/spacer/ORF123/3′ UTR. Thus, the tag/spacer-coding sequence 5′-GCCACCATGGGCCATCACCATCACCATCACAATAGCGCCGTCGACGAACAAAAACTCATCTCAGAAGAGGATCTGAATAGCCATACCGGT3′ was synthesized as an in-frame fusion with a sequence corresponding to CyHV-3 genome co-ordinates 216,593–217,478 inclusive (i.e. from the second codon of *ORF123* to the base immediately upstream of the transcription termination signal). The above DNA sequence was directionally cloned between the HindIII and Xho1 sites of the expression vector pcDNA3.1(+) (Life Technologies). pLS-123-3′ UTR contained the 3′ UTR from CyHV-3 *ORF122/ORF123* inserted immediately downstream of the luciferase coding sequence in the 3′ UTR reporter vector pLightSwitch-3′ UTR (SwitchGear Genomics). This construct was generated by synthesizing and directionally cloning a 67 bp fragment (corresponding to CyHV-3 genome co-ordinates 217,412–217,478 inclusive) between the Xba1 and Xho1 sites of pLightSwitch-3′ UTR. A corresponding plasmid, pLS-123-3′ UTRmut, was also made in which the MR5057-miR-3p seed region match of the predicted miRNA target site (5′GCAATT3′) had been eliminated (changed to 5′CGTACA3′). Constructs were verified by DNA sequencing. Transfections were done using Lipofectamine 2000 (Life Technologies) according to the manufacturer. A custom synthesised MR5057-miR-3p mimic [5′-AAAUUGCGGCCGCUGUCGACGA-3′; Qiagen (miScript miRNA Mimics)] and the Sigma MISSION miRNA Negative Control 1 (HMC0002) were used in transfections (Lipofectamine 2000; Life Technologies). Following transfection, HEK293 cells were processed and analysed for luciferase activity using LightSwitch Luciferase Assay Reagent (Switchgear Genomics, LS010) according to the manufacturer. Statistical analysis was done using the two-tailed Welch's t-test [68]. Western blots were performed as described elsewhere [69] using Anti-MYC tag antibody (Origene Technologies, TA150121) and anti-β-actin antibody (Abcam, A1978).

## Results

### *De Novo* prediction of pre-miRNA-coding sequences on the CyHV-3 genome

In order to investigate its miRNA-coding potential, the CyHV-3 genome (Gen Bank Ac. DQ657948.1) was first analysed using VMir. Six other viral genomes known to collectively encode 94 pre-miRNAs were also analysed so as to establish a set of relevant minimum cut-off values to be used for filtering out predictions that were less likely to represent genuine CyHV-3 pre-miRNAs. Main-Hairpins (MHPs) corresponding to 92 of these known pre-miRNAs were identified using this approach (Dataset B in S1 File). Prediction statistics from these six viral genomes were comparable to those from the CyHV-3 genome and two other viral genomes that have been deemed unlikely to encode pre-miRNAs (Dataset C in S1 File and Dataset D in S1 File). The application of relevant filtering criteria (Minimum MHP size: 50 nt, Minimum Score: 123 and Minimum Window Count: 39; see Dataset E in S1 File) resulted in the elimination of >99% of all predictions from viral genomes analysed, although it still permitted the correct identification of 71/92 known pre-miRNAs. Consequently, 236 CyHV-3 pre-miRNA predictions (excluding repeats) remained after filtering (Table 1). Taking CyHV-3 genome size into account, this represented 0.80 CyHV-3 MHPs per kilobase (kb), this was similar to that of the 6 other viral genomes known to encode pre-miRNAs which had a mean value of 0.76 MHPs per kb post-filtering (Table 1). These values were notably different to those from two other viral genomes predicted to lack pre-miRNAs, which showed a mean value of 0.47 MHPs per kb when analysed using the same criteria (Table 1). Furthermore, out of the remaining 226 CyHV-3 pre-miRNA predictions, 164 (69.5%) of them occurred outside protein-coding regions (i.e. excluding introns) (Dataset F in S1 File) where pre-miRNA genes are more likely to reside [5,6]. In summary, this data gave a strong indication that the CyHV-3 genome had the potential to encode pre-miRNAs.

**Table 1:** VMir pre-miRNA predictions made from herpesvirus genomes.

Pre-miRNA coding potential	Viral Genome^a^	Predicted MHPs post-filtering^b^	% Reduction in predicted MHPs post-filtering	Filtered MHP sites per Kb post-filtering	Correctly predicted known miRNAs post-filtering
Genomes known to encode pre-miRNAs	EBV	124	99.54	0.67	25/25
	HSV-1	160	99.32	1.05	6/15
	HCMV	206	99.40	0.87	10/11
	KSHV	91	99.53	0.66	11/13
	MDV-1	118	99.50	0.66	10/14
	MGHV-68	75	99.54	0.63	9/14
	**Mean**	**129**	**99.47**	**0.76**	**N/A**
Genomes deemed unlikely to encode pre-miRNAs	HHV-3^c^	56	99.67	0.45	N/A
	HHV-7^c^ ^,^ ^d^	76	99.56	0.50	N/A
	**Mean**	**66**	**99.62**	**0.47**	**N/A**
Genome under Investigation	CyHV-3	236	99.46	0.80	N/A

^a^Abbreviations: EBV—Epstein-Barr virus, HSV-1—Herpes Simplex-1, HCMV-Human Cytomegalovirus, KSHV—Kaposi’s sarcoma-associated herpesvirus, MDV-1—Marek’s Disease virus Type 1, MGHV68—Mouse Gammaherpesvirus 68, HHV-3-Human herpesvirus type 3 (also known as Varicella Zoster virus), HHV-7-Human herpesvirus 7.

^b^ Filters applied: Min. MHP size -50, Min. Score -123, Min. Relative-WC -39.

^c^ This viral genome was predicted elsewhere not to encode pre-miRNAs [28]

^d^ Deep sequencing of small RNAs from tissue infected with this virus failed to detect any virus encoded miRNAs [70]

### Identification of high-probability pre-miRNAs following deep sequencing of small RNAs from *in vitro* CyHV-3 infections

**Fig 1:**
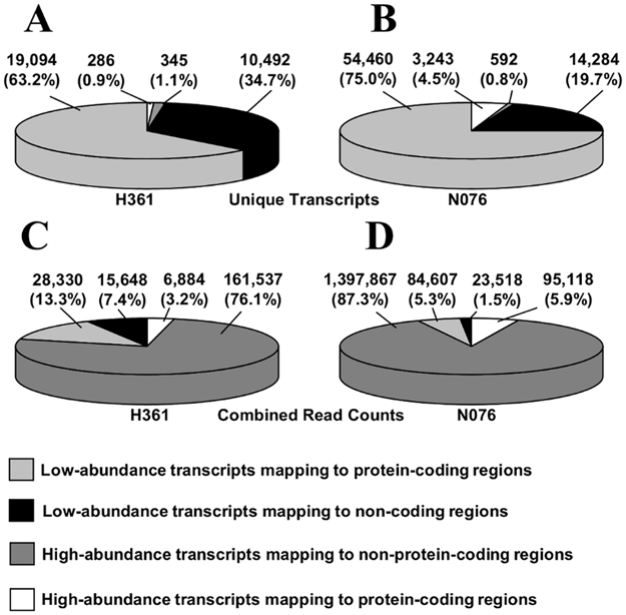
Small RNA sequence reads derived from CyHV-3 during *in vitro* infection of CCB cells. **(A)** and **(B)** The numbers and general genomic locations of low- and high abundance unique viral reads determined following deep sequencing of small RNAs isolated from CyHV-3-infected CCB cells using viral isolates H361 and N076. **(C)** and **(D)** Analysis of the combined read counts of low- and high-abundance unique CyHV-3 reads sequenced from both infections.

In order to experimentally determine if CyHV-3 encoded its own miRNAs, productive/lytic infections of CCB cells were first set up using the two CyHV-3 isolates H361 and N076. Size-selected small RNAs (17–25 nt) were then prepared from infected cells and analysed by deep sequencing, yielding a total of 12,016,246 (H361) and 23,903,786 (N076) small RNA sequence reads. All unique sequence tags obtained from both infections are available for download via the Dryad Digital Repository (http://dx.doi.org/10.5061/dryad.3c7m4). CyHV-3-derived reads accounted for 1.8% (212,399) and 6.7% (1,601,110) of the total from the H361 and N076 infections, respectively. SeqMonk coverage plots of all reads mapping to the CyHV-3 genome showed that most of the CyHV-3 genome was transcribed and that there was a high degree of congruency between the two infections in terms of both the qualitative and quantitative profiles observed (S1 Fig). The 17–25 nt RNA samples were expected to be specifically enriched for miRNAs and indeed the most abundant small RNAs present were found to be evolutionarily conserved host miRNAs. Similarly, CyHV-3 encoded miRNAs were expected to be among the most abundant viral reads and thus those with read counts ≥10 (median read count was 1) were classified as highly abundant and therefore of particular interest. As CyHV-3 pre-miRNAs were more likely to be derived from non-protein-coding regions, only abundant reads that mapped to such loci were considered initially. Although, such reads only represented a small subset of unique CyHV-3 reads (Fig 1A and 1B) they actually accounted for the vast majority of CyHV-3 reads, in terms of total read counts (Fig 1C and 1D).

**Fig 2:**
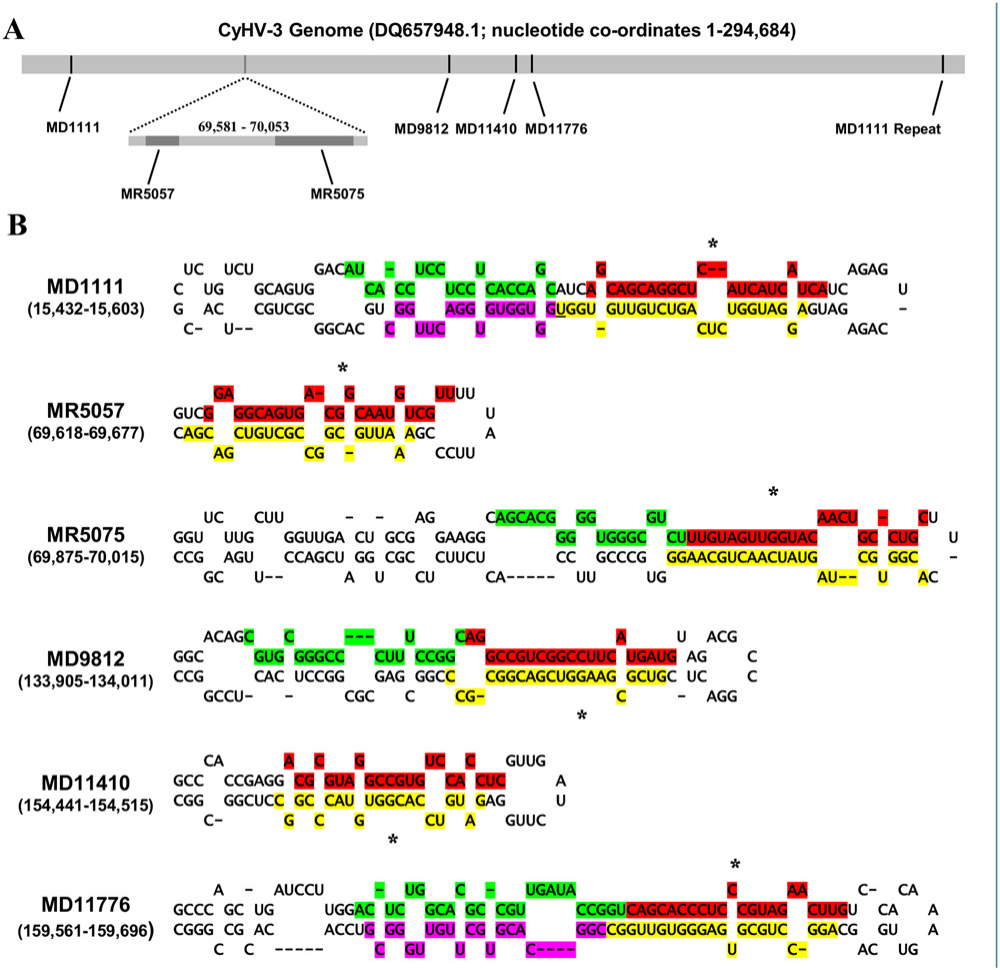
Genomic locations and structures of high probability CyHV-3 pre-miRNA identified using a non-automated approach. **(A)** Genomic locations of pre-miRNAs; all features on the forward strand are shown in black and those on the reverse strand are in grey. **(B)** Structures (predicted by VMir) of the high-probability CyHV-3 pre-miRNAs shown in (A). The 5′/3′ arm miRNAs and 5′/3′ arm moRNAs are highlighted in red/yellow and green/purple respectively and represent the dominant reads at these loci in the H361 infection, variants (if any) identified in the N076 infection are given in Table 2. MiRNA* reads are indicated with an asterisk. MiRNAs partially overlapping with moRNAs are underlined.

Taking a non-automated approach, reads were first cross-referenced with the output from VMir. This resulted in the identification of 21 pre-miRNA-like candidates (Dataset G in S1 File) that complied with points (i)-(iv) of the pre-miRNA criteria applied in this study, however only six of these loci also complied with points (v)-(ix). The evidence suggested that these six candidates could give rise to twelve mature miRNAs, with further indications that four of them could also give rise to at least 7 potential moRNAs (Fig 2 and Table 2). With the exception of pre-miRNA candidate MD1111, the miRNA/miRNA* patterns remained the same in both H361 and N076 infections in terms of the pre-miRNA arms from which they were derived. The sequences of 9/12 of these putative miRNA/miRNAs* were identical in both infections, and of the three that differed, only one showed a change at the 5′ end (differing by 1 nt) (Table 2). In contrast only 13/30 sequences representing putative miRNAs from the 15 low-probability pre-miRNAs were identical in both infections, and 10/30 were different at their 5′ ends, in most cases differing by more than 1 nt (Dataset H in S1 File). All high-probability pre-miRNAs were classified as ‘real’ by both pre-miRNA classifiers (MiPred and CHSMM) and had MFE values <−25 kcal/mol that were statistically significant (p <0.05) (Dataset I in S1 File), a feature which is key in distinguishing between pre-miRNA-like and non-pre-miRNA-like hairpins [54,71]. In contrast only 4/15 low-probability pre-miRNAs were classified as real by both classifiers and 6/15 did not have MFE values <−25 kcal/mol (Dataset I in S1 File).

**Table 2:** Details of putative CyHV-3 miRNAs/moRNAs from high-probability pre-miRNAs using a non-automated approach.

Pre-miRNA candidate	Arm ^a^	CyHV-3 isolate	Sequence (5′ to 3')	Read Count ^b^	Read present in both infections	Same read designated miRNA/moRNA in both infections
MD1111	-miR-5p	H361	AGCAGCAGGCUCAUCAUCAUCA	567*	Yes	Yes
		N076	AGCAGCAGGCUCAUCAUCAUCA	1055	Yes	
	-miR-3p	H361	AGGAUGGUCUCAGUCUGUUGUGGU	2711	Yes	Yes
		N076	AGGAUGGUCUCAGUCUGUUGUGGU	72*	Yes	
	-moR-5p	H361	AUCACCUCCUCCUCACCAGC	3	No	No. 5′ end offset +/- 1nt
		N076	UCACCUCCUCCUCACCAGC	4	No	
	-moR-3p	H361	UGGUGGUGUGGACUUGGC	38	Yes	No. Same 5′ End
		N076	UGGUGGUGUGGACUUGGCU	85	Yes	
MD9812	-miR-5p	H361	AGGCCGUCGGCCUUCAUGAUG	23	Yes	No. Same 5′ End
		N076	AGGCCGUCGGCCUUCAUGAUGU	7	Yes	
	-miR-3p	H361	GUCGCGAAGGUCGACGGCGCC	1*	Yes	Yes
		N076	GUCGCGAAGGUCGACGGCGCC	5*	Yes	
	-moR-5p	H361	CGUGCGGGCCCUUUCCGGC	1	Yes	Yes
		N076	CGUGCGGGCCCUUUCCGGC	1	Yes	
	-moR-3p	H361	N/A	N/A	N/A	N/A
		N076	CGGCGAGCGCGGCCUCACUCC	1	N/A	
MD11410	-miR-5p	H361	ACGCGUAGGCCGUGUCCACCUC	10	Yes	Yes
		N076	ACGCGUAGGCCGUGUCCACCUC	26	Yes	
	-miR-3p	H361	GAUGUCCACGGUGUACCCGGC	4*	No	No. 5′ end offset +/- 1nt
		N076	AUGUCCACGGUGUACCCGGCC	2*	No	
MD11776	-miR-5p	H361	CAGCACCCUCCCGUAGAACUUG	301*	Yes	Yes
		N076	CAGCACCCUCCCGUAGAACUUG	1126*	Yes	
	-miR-3p	H361	AGGCCUGCGUGAGGGUGUUGGC	2114	Yes	No. Same 5′ End
		N076	AGGCCUGCGUGAGGGUGUUGG	10396	Yes	
	-moR-5p	H361	ACUCUGGCACGCCGUUGAUACCGGU	18	Yes	No
		N076	UCUGGCACGCCGUUGAUACCGGU	85	Yes	
	-moR-3p	H361	CGGCACGUGCUUGUUGGGCG	60	Yes	Yes
		N076	CGGCACGUGCUUGUUGGGCG	3171	Yes	
MR5057	-miR-5p	H361	GGAGGCAGUGACGGCAAUGUCGUU	15*	Yes	Yes
		N076	GGAGGCAGUGACGGCAAUGUCGUU	149*	Yes	
	-miR-3p	H361	AAAUUGCGGCCGCUGUCGACGA	62596	Yes	Yes
		N076	AAAUUGCGGCCGCUGUCGACGA	1166058	Yes	
MR5075	-miR-5p	H361	UUGUAGUUGGUACAACUGCCUGC	12*	Yes	Yes
		N076	UUGUAGUUGGUACAACUGCCUGC	95*	Yes	
	-miR-3p	H361	ACGGUGCUAGUAUCAACUGCAAGG	125	Yes	Yes
		N076	ACGGUGCUAGUAUCAACUGCAAGG	217	Yes	
	-moR-5p	H361	AGCACGGGGGUGGGCGUCU	26	Yes	Yes
		N076	AGCACGGGGGUGGGCGUCU	122	Yes	

^a^ MiRNAs and moRNAs are differentiated using the prefix miR and moR respectively.

^b^ MiRNA* reads counts are denoted with an asterisk after the corresponding read count.

Assessment of the small RNA alignment signatures mapping to all 21 candidates revealed a stark contrast between high and low-probability pre-miRNAs. In both infections, low-probability pre-miRNAs (except MR6201) had signatures that were not consistent with the currently accepted model of miRNA biogenesis [56], and instead consisted mainly of randomly overlapping reads, offset across the predicted pre-miRNA sequence (Dataset J in S1 File). In contrast, the high-probability pre-miRNAs and MR6201 showed strong miRNA-like alignment signatures in both infections with groups of reads arranged into individual “stacks” representing either putative miRNAs or moRNAs (and associated isomiRs/isomoRs). The alignment signature for MD11776 was particularly consistent with the ideal miRNA alignment signature. In addition, transcripts were observed from five distinct regions of the MD11776 locus, representing the two miRNAs, two moRNAs and a terminal loop sequence resembling what is described as a “five-phased-precursor” [20,72,73] (Dataset J in S1 File). MR6201 was the only low-probability pre-miRNA locus to display a miRNA-like alignment signature, however reads mapping to this and other low-probability pre-miRNA loci did not form discrete read stacks or discrete regions of read enrichment relative to flanking genomic regions (Dataset K in S1 File). In contrast, such features were present in both infections at all six high-probability pre-miRNA loci (Dataset K in S1 File), with the exception of reads mapping to MD11410 in the N076 infection. However, despite not being enriched, these reads derived from MD11410 were nonetheless present in greater numbers than those derived from flanking genomic regions (Dataset K in S1 File). Although located on non-coding parts of the genome, all fifteen low-probability pre-miRNA loci in fact mapped to 3′ UTRs of upstream ORFs. This suggested that some of their associated reads, which often extended beyond the boundaries of pre-miRNA loci, were in fact derived from degraded protein-coding transcripts that were initiated further upstream (Dataset K in S1 File).

In contrast to the practical limitations associated with the non-automated approach to miRNA identification, the use of an automated approach, in this case a combination of miRDeep2 and MIREAP, facilitated the analysis of the entire deep sequencing dataset. Thus, this potentially allowed the identification of other viral pre-miRNAs that produced miRNAs of lower abundance and/or that mapped to within protein-coding regions. Out of a total of 76 potential pre-miRNAs identified using this automated approach (results detailed in: Dataset L in S1 File, Dataset M in S1 File, Dataset N in S1 File, Dataset O in S1 File, Dataset P in S1 File), only seven of them were independently identified by both programmes, and of these, only one (MR17383) was located in a protein-coding region, however it was not classified as a real pre-miRNA by either of the pre-miRNA classifiers. Interestingly, of the remaining six, five had also earlier been classified as high-probability pre-miRNAs using the non-automated approach (Table 3). The same five also represented the top ranked miRdeep2 intergenic pre-miRNA candidates from both infections, based on miRdeep2 scores (Dataset L in S1 File). The fact that both the non-automated and automated approaches were largely in agreement supports the criteria used to eliminate low-probability pre-miRNA candidates in both cases. The only other pre-miRNA candidate from a non-coding region to appear in multiple sets of results was MD11704 (Dataset M in S1 File). Although it occurs near MD11776 (<1 kb, Dataset O in S1 File) and largely complied with the pre-miRNA identification criteria, it was only identified by MIREAP alone (Table 3).

**Table 3:** Summary of high-probability CyHV-3 pre-miRNAs Identified using both approaches.

Pre-miRNA candidate ^a^	Pre-miRNA candidates from non-coding regions identified by both programs or multiple times by the same program (automated approach)				High probability pre-miRNA candidate(non-automated approach)	High probability CyHV-3 pre-miRNA
	miRDeep2		MIREAP			
	H361 Isolate	N076 Isolate	H361 Isolate	N076 Isolate		
MR5057	Yes	Yes	Yes	Yes	Yes	Yes
MD11776	Yes	Yes	Yes	Yes	Yes	Yes
MD11410	Yes	Yes	Yes	No	Yes	Yes
MD9812	Yes	No	Yes	Yes	Yes	Yes
MD1111	Yes	No	Yes	No	Yes	Yes
MR6201^b^	Yes	No	Yes	No	No	No
MD11704 ^b^	No	No	Yes	Yes	No	No
MR5075	No	No	No	No	Yes	Yes

^a^ Pre-miRNA candidates are referred to using IDs originally assigned to the same predicted hairpin structures by VMir.

^b^ Classified as putative CyHV-3 pre-miRNA due to non-compliance with Points (ii) and/or (ix) of pre-miRNA identification criteria.

In summary, using both approaches, a total of 8 high probability pre-miRNA candidates were identified from the deep sequencing data (Table 3). Although MR6201 was independently identified by both automated methods, based on results from the non-automated approach, it remains possible that reads from this locus were in fact degraded fragments of the ORF50 mRNA 3′ UTR (Dataset K in S1 File). Notably, reads mapping to the MD11704 loci were too low in read count (Dataset O in S1 File) to be considered a high-probability candidate in this study. For these reasons, these loci are therefore classed as putative pre-miRNAs until further experimental evidence suggests otherwise. Ultimately, six of the candidates in Table 3 were scored as high-probability CyHV-3 pre-miRNAs as they fully complied with pre-miRNA identification criteria.

MiRNAs from the six high-probability pre-miRNAs were also accompanied by isomiRs (Dataset J in S1 File). As isomiRs generally display a greater degree of 3′ end heterogeneity [74–77] we therefore compared the extent of 3′ versus 5′ end heterogeneity within each isomiR. This analysis revealed that (i) most isomiR sets were comprised mainly of isomiRs offset at the 3′ end (Ratio-1), (ii) isomiR sets consisted of isomiRs that, on average, had greater 3′ end offsets (measured in nts) (Ratio-2) and (iii) most isomiR sets had greater numbers of isomiRs offset at the 3′ end, indicating that 5′ end processing was generally more consistent (Ratio-3). Overall, these results indicated a general bias towards greater 3′ end heterogeneity within individual isomiR sets (Table 4; Dataset Q in S1 File and Dataset R in S1 File).

**Table 4:** IsomiR end heterogeneity analysis for miRNAs from high-probability CyHV-3 pre-miRNAs.

End heterogeneity among isomiR sets	Ratio-1: Number of Unique 3′ End: 5′ End offset isomiRs	Ratio-2: 3′ End: 5′ End average absolute offset	Ratio-3 3′ End: 5′ End processing consistency
% of isomiR sets displaying 3′ bias	58.3%	87.5%	66.7%
% of isomiR sets displaying 5′ bias	8.3%	4.2%	25.0%

### Detection of CyHV-3 miRNAs by DNA microarray analysis

**Fig 3:**
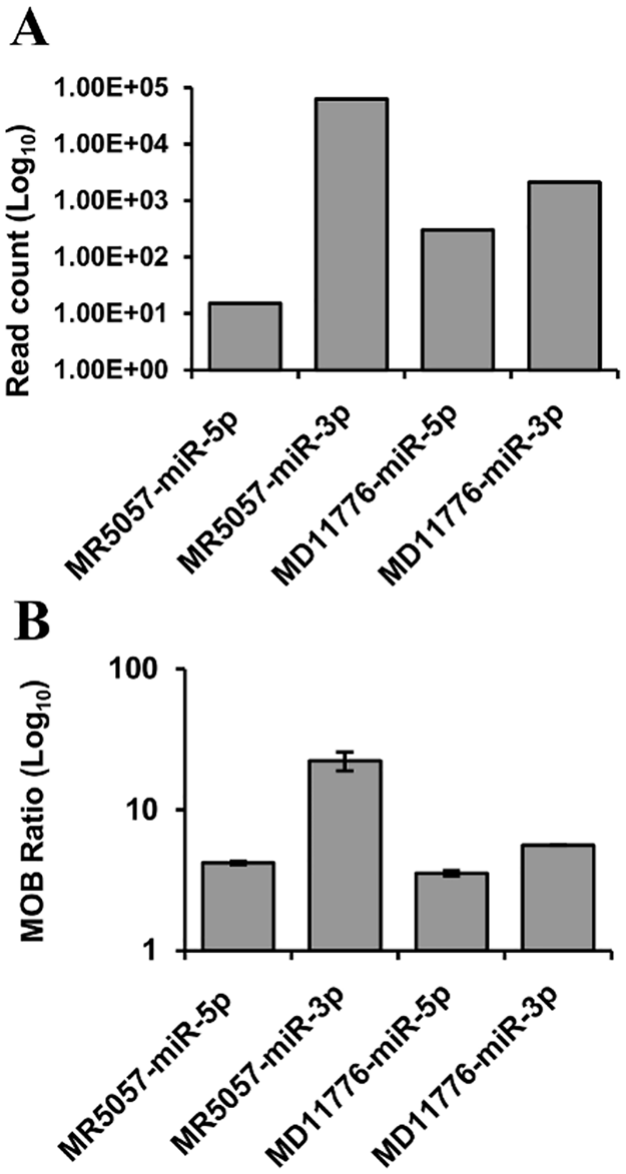
Comparison of the levels of miRNAs derived from CyHV-3 pre-miRNA candidates MR5057 and MD11776. (A) Small RNA deep sequencing read counts and (B) DNA microarray miRNA probe hybridization signal intensities corrected and expressed as multiples of background (MOB). Data from both (A) and (B) were obtained using size-selected miRNA-inclusive RNA (F1 fraction; 17–25 nt) from the same *in vitro* infection (CyHV-3 H361 isolate). No corresponding hybridization signals were detected in miRNA non-inclusive RNA (F2 faction; 26–35 nt) from the same infection or in miRNA inclusive RNA (F3 fraction, 17–25 nt) from non-infected cells (Dataset S in S1 File). Error bars represent the data range.

The same RNA from the H361 infection was used in a DNA microarray hybridization experiment. Size-selected small RNAs from both miRNA-inclusive (17–25 nt) and non-inclusive (26–35 nt) size ranges (referred to as F1 and F2 respectively) were thus analysed using probes targeting putative miRNAs from both high and low-probability pre-miRNAs. MiRNA-inclusive size selected RNA (F3) from non-infected CCB cells was also analysed as a negative control. Known host miRNAs were detected in F1 and F3 but not in F2 and an exogenous spike miRNA control was detected in all samples (Dataset S in S1 File). Putative miRNAs mapping to two out of the six high-probability pre-miRNAs (MR5057 and MD11776) gave positive signals in F1 but not in F2 or F3 (Dataset S in S1 File). MiRNAs from both arms of these two pre-miRNAs were detected and their relative levels generally concurred with those determined from deep sequencing, although this data suggested that MR5057-miR-5p was present at higher levels than MD11776-miR-5p (Fig 3). Notably, 10/15 low-probability pre-miRNAs identified using the non-automated approach gave positive signals in F1 but 12/15 showed positive signals in F2 implying that they were degradation products of overlapping transcripts, most likely 3′ UTRs (Dataset S in S1 File).

DNA microarray analysis was also used to test if ‘highly abundant’ small RNAs were in fact merely over-represented in the data due to the enzymatic bias. The small RNAs derived from all pre-miRNA candidates (Dataset G in S1 File) were among the most abundant identified in the deep sequencing data from the H361 infection. The 12 of these that were detected in F1 also gave some of the strongest hybridization signals with an average percentile rank of 97 (Dataset S in S1 File). As the hybridization samples were processed in the absence of any enzymatic bias, this verified that most of these were indeed highly abundant within the sample. Other VMir miRNA predictions in which related small RNA read counts may have been under-represented or absent due to enzymatic bias were also examined. Although miRNA-like signals (i.e. F1 +ve, F2 −ve and F3 −ve) were identified among these, it was found that either the corresponding sequencing data and/or predicted pre-miRNAs at such loci displayed gross non-compliance with the pre-miRNA identification criteria or the signals were caused by array surface artefacts. In summary, the DNA microarray data agreed well with the deep sequencing output regarding two high-probability pre-miRNAs (MR5057 and MD11776). It also validated the criteria that were applied to eliminate low-probability pre-miRNA candidates following deep sequencing. Furthermore, deep sequencing data did not corroborate with DNA microarray evidence regarding the existence of additional potential CyHV-3 pre-miRNAs.

### Detection of MR5057-miR-3p by Northern Blotting in CyHV-3-infected CCB cells

**Fig 4:**
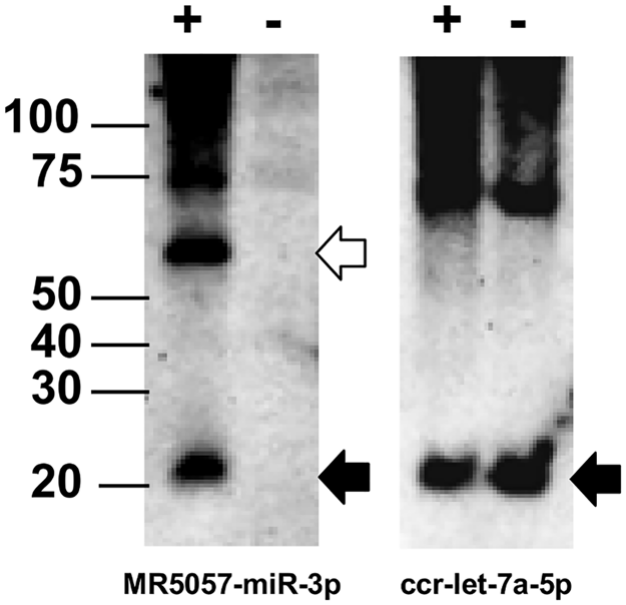
Detection of CyHV-3 miRNA MR5057-miR-3p and its pre-miRNA by Northern blotting. Total RNA was prepared from CCB cells following an *in vitro* lytic infection with the CyHV-3 N076 isolate (+) and from uninfected control cells (-). Replicate blots were probed separately for MR5057-miR-3p (left), and the host miRNA ccr-let-7a-5p which served as a loading control (right). The location of size markers (in nucleotides) is indicated on the left; black arrows point to bands corresponding to MR5057-miR-3p and ccr-let-7a-5p (both 22 nt); the open arrow points to the band corresponding to the MR5057 pre-miRNA (56 nt).

Expression of the four most abundant miRNAs was also investigated by Northern blotting. Total RNA was prepared at 12 dpi from a second *in vitro* lytic infection using the CyHV-3 N076 isolate and also from uninfected CCB cells as control. Hybridization using a probe for MR5057-miR-3p resulted in the detection of two discrete transcripts in RNA prepared from the CyHV-3 infection but not from the uninfected control (Fig 4). These corresponded to the expected sizes for the mature miRNA (22 nt) identified by deep sequencing and the pre-miRNA MR5057 (56 nt) produced after Drosha processing (size inferred from the positions of the 5′ end of MR5057-miR-5p and the 3′ end of MR5057-miR-3p). Detection of the host miRNA ccr-let-7a confirmed that RNA quality was comparable in both samples. This experiment verified that MR5057-miR-3p occurs as a fully processed miRNA from CyHV-3 pre-miRNA MR5057. Using the same approach, we were unable to detect MD11776-miR-3p, MD1111-miR-3p and MR5075-miR-3p or CyHV-3-specific bands corresponding to the expected size of their respective pre-miRNAs.

### Detection of CyHV-3 miRNAs by Stem-Loop RT-qPCR following productive CyHV-3 infections *in vitro* and *in vivo*

**Fig 5:**
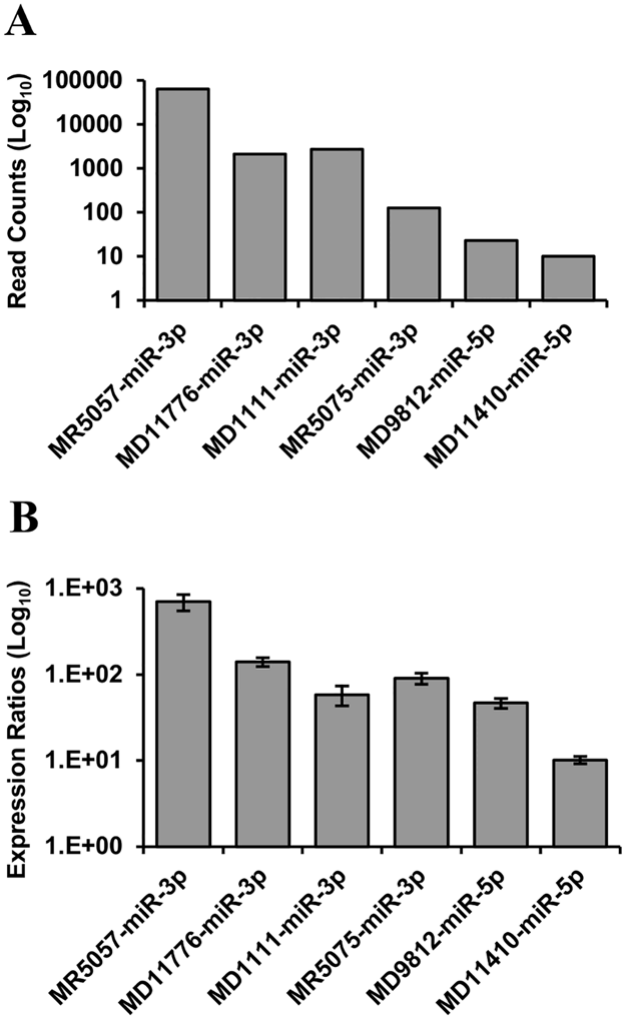
Relative expression levels of the dominant miRNAs from six high probability CyHV-3 pre-miRNAs. RNA prepared from CyHV-3-infected CCB cells was analysed for viral miRNA expression by **(A)** small RNA deep sequencing (read counts), and **(B)** stem-loop RT-qPCR. In (B), all input cDNA came from a common RT reaction; values shown are based on means of replicates and error bars represent the data range.

**Fig 6:**
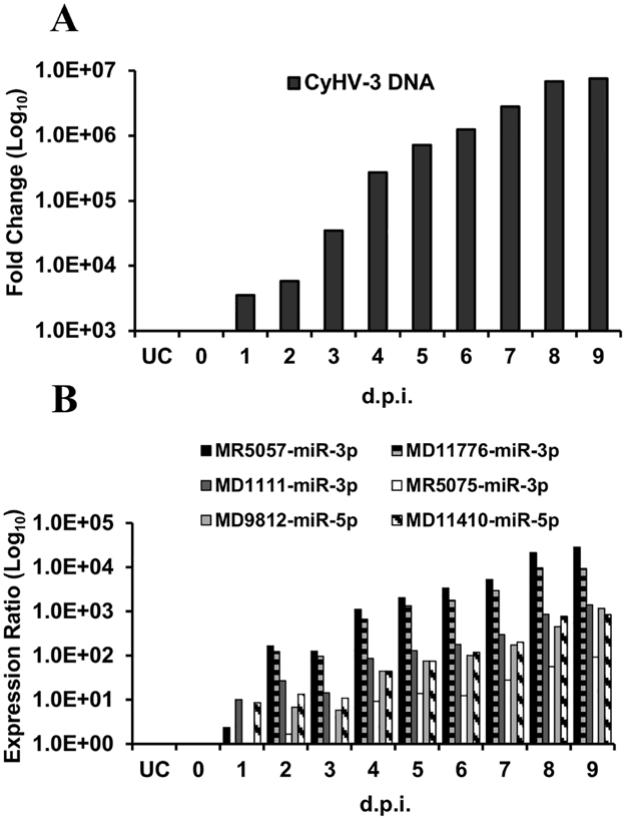
CyHV-3 miRNAs are consistently expressed during *in vitro* infection of CCB cells. The numbers underneath each graph indicate days post-infection (d.p.i.) with the CyHV-3 N076 isolate. UC denotes uninfected cells. **(A)** Detection of CyHV-3 genomic DNA by PCR; levels were normalized to CCB cell genomic DNA in each case. **(B)** Relative levels of the dominant miRNAs from six high probability CyHV-3 pre-miRNAs over the course of the experiment shown in (A) as measured by stem-loop RT-qPCR and normalized to levels of the host miRNA ccr-let-7a.

**Fig 7:**
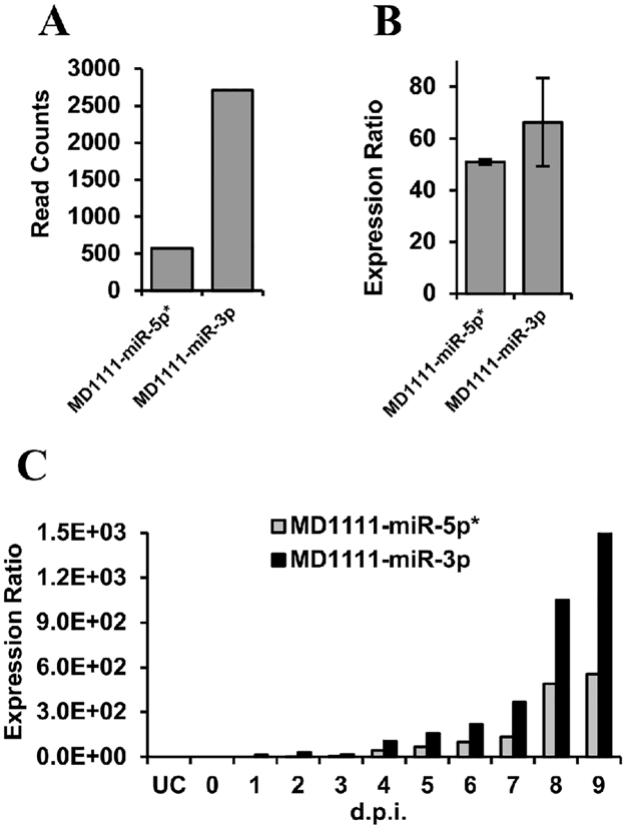
Relative levels of miRNAs from both arms of CyHV-3 pre-miRNA MD1111. **(A)** Small RNA deep sequencing read counts obtained following infection of CCB cells with CyHV-3 isolate H361, and **(B)** stem-loop RT-qPCR analysis of RNA from same *in vitro* infection. Assays were done in duplicate; values shown are based on the means of replicates (error bars represent the data range). **(C)** Stem-loop RT-qPCR analysis of total RNA from a second non-synchronous *in vitro* infection (CyHV-3 N076 isolate). Relative expression levels were normalized to host cell miRNA ccr-let-7a. All results are in agreement that MD1111-miR-5p is the miRNA*.

MiRNAs often escape detection using hybridization technologies that do not involve pre-amplification of the target sequence. Stem-loop RT-qPCR is a sensitive technique for the specific detection of mature miRNAs [78] and it was therefore used to re-test the same RNA from the H361 infection used for deep sequencing. Custom stem-loop RT-qPCR assays were designed to detect the dominant miRNAs from the 6 high-probability CyHV-3 pre-miRNAs. Using these assays, all target miRNAs were detected in the H361 sample and the hierarchy in expression levels was generally comparable to that observed in the deep sequencing data (Fig 5). The same stem-loop RT-qPCR assays were then used to analyse RNA sampled over the course of a second *in vitro* non-synchronous infection (N076 isolate, biological replicates representing such infections at 0–9 dpi). The levels of CyHV-3 DNA were seen to increase over the sampling period (Fig 6A) and corresponding increases in the levels of each CyHV-3 miRNA was also observed, all of which were detectable by 2 dpi (Fig 6B). The hierarchy in miRNA levels was consistent with the observations from the H361 infection (Fig 5), with the exception of MR5075-miR-3p, which was present at lower levels relative to other targets (Fig 6B). In order to investigate discrepancies between the two sequencing experiments regarding the miRNA/miRNA* pattern from MD1111 (Table 2), stem-loop RT-qPCR was used to measure the levels of both MD1111 miRNAs in the same samples described above. The data was consistent, indicating that the MD1111 miRNA* was in fact derived from the 5′ arm in accordance with the deep sequencing data from the H361 infection (Fig 7).

**Fig 8:**
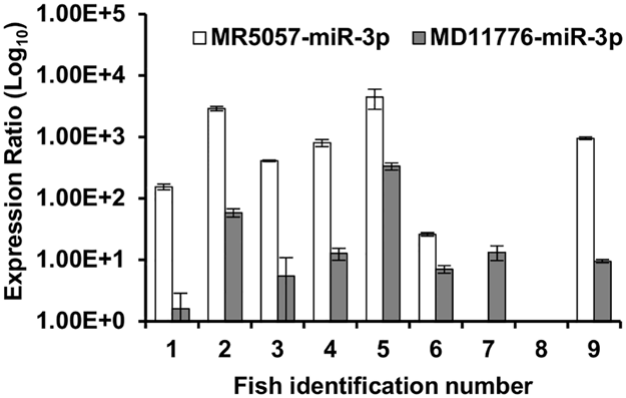
MiRNAs MR5057-miR-3p and MD11776-miR-3p are expressed in gill tissues from CyHV-3-infected carp. Total RNA was extracted from samples of gill tissue taken from both healthy carp and carp with Koi Herpesvirus Associated Disease (KHVD), and were analysed for CyHV-3 miRNAs by stem-loop RT-qPCR. Relative values were normalized to levels of host cell miRNA ccr-let-7a. Assays were done in triplicate, values shown are based on means of replicates and error bars represent the standard deviation.

We next used stem-loop RT-qPCR to confirm expression of MR5057-miR-3p and MD11776-miR-5p *in vivo*. Total RNA was extracted from carp gill tissue samples originating from sites of suspected KHVD outbreaks in the UK. All fish were shown to be CyHV-3 positive by both virus isolation and PCR (Dataset T in S1 File). MR5057-miR-3p and MD11776-miR-5p were detected in 7 and 8 out of the 9 samples, respectively (Fig 8). The target viral miRNAs were not detected in gill tissue from 5 CyHV-3-negative carp, taken from KHVD free sites and processed at the same time as positive samples (Dataset T in S1 File). MiRNA integrity was confirmed in all samples by ccr-let-7a analysis (Dataset T in S1 File). As per observations *in vitro*, MR5057-miR-3p was expressed at higher levels than MD11776-miR-3p, with the exception of sample 7, where MD11776-miR-3p was detected in the absence of MR5057-miR-3p (Fig 8).

### Identification of CyHV-3 pre-miRNA homologues in other CyHVs and potential miRNA functional homologues

**Fig 9:**
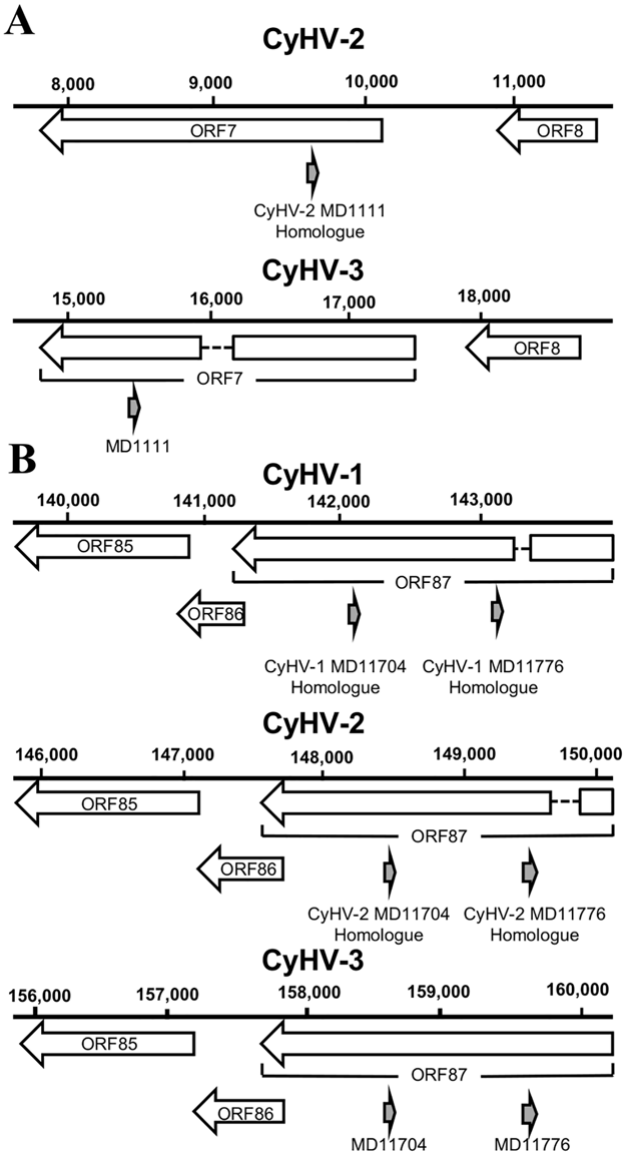
Identification of CyHV-3 pre-miRNA homologues on the genomes of CyHV-1 and -2. Genomic locations of **(A)** CyHV-3 pre-miRNA MD1111 relative to its potential homologue on the CyHV-2 genome and **(B)** CyHV-3 pre-miRNAs MD11776 and MD11704 relative to their potential homologues on the CyHV-1 and CyHV-2 genomes.

**Fig 10:**
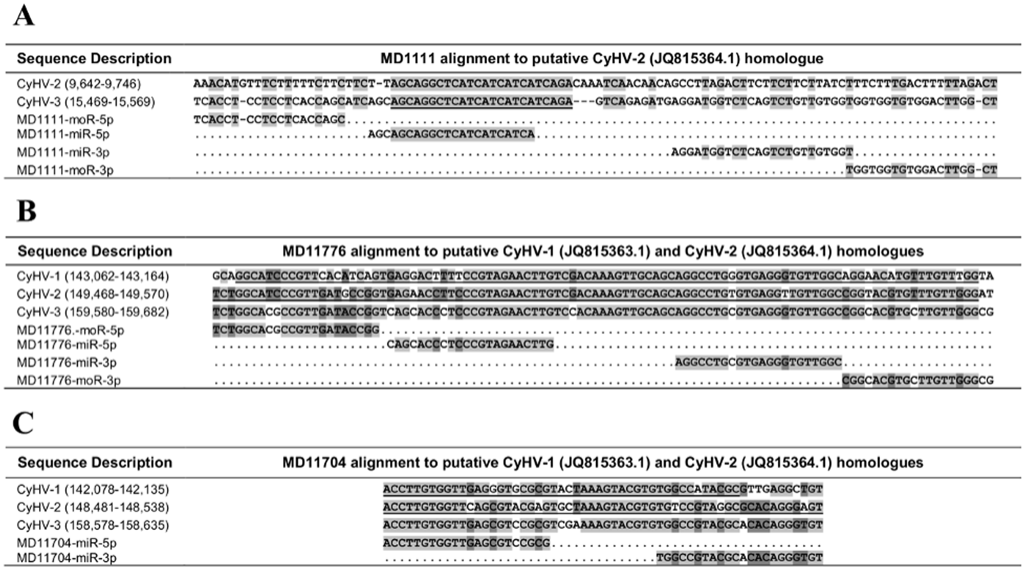
Alignment of CyHV-3 pre-miRNAs to candidate homologues on the CyHV-1 and CyHV-2 genomes. CyHV-1 and CyHV-2 sequences that are underlined represent homologous sequences identified by BLASTN, sequences not underlined (i.e. remainder of input sequences) were subsequently manually inspected for sequence similarity. Nucleotides shaded in grey are conserved in all viruses in the respective alignments and those in dark grey are conserved in 2/3 viruses. **(A)** MD1111—with the exception of the first 3 nucleotides, the MD1111-miR-5p sequence is fully conserved in CyHV-2; **(B)** MD11776—the seed (bases 2–7) of MD11776-miR-3p is fully conserved and the remainder of the miRNA is also conserved in all three viruses with the exception of two mismatches; **(C)** MD11704—the MD11704-miR-5p seed region is fully conserved, the MD11704-miR-3p seed region has one mismatch to each virus.

We used BLASTN [62] to align high-probability and putative CyHV-3 pre-miRNA sequences to the genomes of the closely related CyHV-1 and CyHV-2 [79,80]. Although many short alignments were identified across both genomes, the best MD1111, MD11776 and MD11704 alignments all consisted of relatively longer sequences (all of which had E-values ≤1.00^–6^, Dataset U in S1 File). Interestingly, these were also the only alignments to occur within corresponding orthologous regions of each viral genome (Fig 9). In addition, the sequence similarity was greatest in the regions encoding the miRNAs and especially within the seed region of MD11776-miR-3p and MD11704-miR-3p (Fig 10). In VMir analysis of the CyHV-1 and CyHV-2 genomes, only the potential MD11776 CyHV-2 homologue corresponded to a predicted pre-miRNA, which in addition was classified as ‘Real’ by both MiPred and CSHMM (Dataset V in S1 File). We also noted that BLASTN alignment of CyHV-3 pre-miRNAs to the genomes of three fully sequenced CyHV-3 strains (KHV-U, KHV-1 and TUMST1) [81] showed that MD1111 and MR5075 are not conserved in the TUMST1 strain, with both of the TUMST1 pre-miRNA homologues containing significant insertions (9 bp in the loop for MD1111 and separate 6 bp and 4 bp insertions in the MR5075-miR-5p sequence).

The seed regions of the CyHV-3 miRNAs were also compared to those of other known viral and host miRNAs in miRBase. Six miRNAs from five of the CyHV-3 pre-miRNAs had either full or partial matches (1 mismatch) to seed regions from other viral miRNAs. Two had full seed matches and one of these (MD11776-miR-5p) had a full match to miRNAs from four different viruses (Dataset W in S1 File). Three CyHV-3 5′ arm miRNAs (all miRNAs*) had matches to multiple host miRNAs, although many multiple matches were to members of the same miRNA families that share the same seed sequence (Dataset X in S1 File).

### Identification of potential mRNA targets for CyHV-3 miRNAs

**Fig 11:**
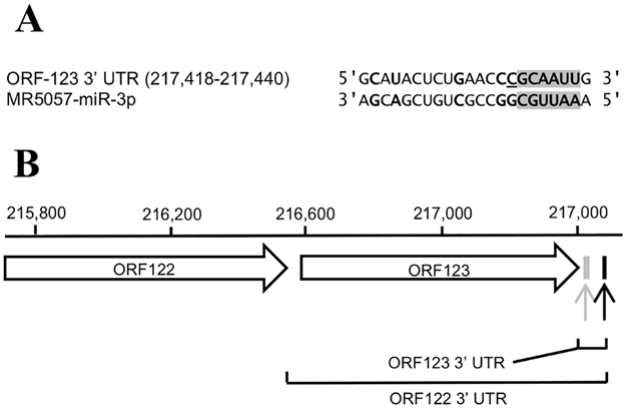
Identification of a potential target for CyHV-3 MR5057-miR-3p. **(A)** Base pairing between MR5057-miR-3p and target site in the *ORF123* 3′ UTR (genomic co-ordinates in brackets). It is a 7mer-m8 site, in line with the nomenclature system outlined elsewhere [65]. Complementary nucleotides are shown in bold. Seed matches are highlighted in dark grey. An additional nucleotide representing part of the 7mer-m8 target site is underlined. **(B)** Genomic location of the same site described in (A) (indicated by the light grey arrow and box). It is located in the 3′ UTR of *ORF123* which itself overlaps with the predicted longer 3′ UTR from *ORF122*. The black arrow and box show the location of the *ORF122/ORF123* polyA signal.

CyHV-3 miRNAs that accumulate at high levels during lytic infections are likely to have roles during that stage of the viral life cycle. As the CyHV-3 miRNA MR5057-miR-3p was consistently found to be present at the highest levels in all experiments, TargetScan and PITA were therefore used to identify candidate MR5057-miR-3p target sites on CyHV-3 mRNAs. TargetScan takes many miRNA target site attributes into account, including sequence pairing within and outside of the seed, seed base-pairing stability and local sequence composition [64–66] whereas PITA ranks potential target sites based entirely on their accessibility [67]. TargetScan identified a single target site for MR5057-miR-3p situated within the 3′ UTR of *ORF122/ORF123* (Context Score = -0.139) (Fig 11). The same site was also deemed to be the most accessible MR5057-miR-3p 3′ UTR target by PITA (ΔΔG = -13.65). MR5057-miR-3p also occurs antisense to CyHV-3 *ORF43*, with which it has full complementary, and was also ranked as the most accessible protein-coding MR5057-miR-3p target site by PITA (ΔΔG = -25.01).

### MR5057-miR-3p targets a sequence in the 3′ UTR of *ORF123* and downregulates the level of its encoded protein

**Fig 12:**
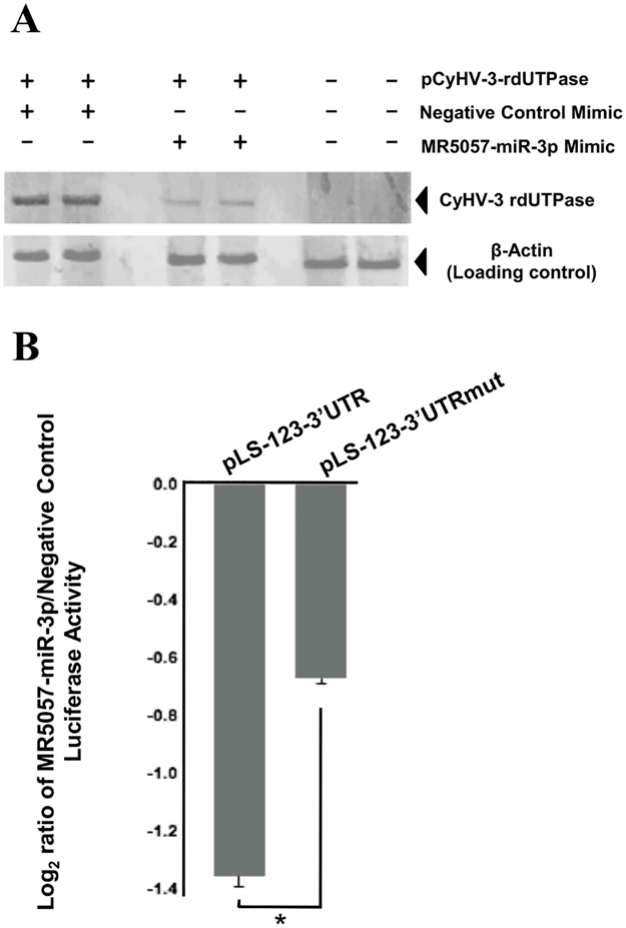
MR5057-miR-3p interacts with the 3′ UTR of CyHV-3 *ORF123* and downregulates its encoded protein. **(A)** Western blot showing relative CyHV-3 rdUTPase levels (top panel) produced 48 h following transfection of CCB cells with pCyHV-3-rdUTPase in combination with miRNA mimics (as indicated above the image). Endogenous β-actin levels are also shown from the same blot (lower panel). **(B)** Luciferase assays showing relative values obtained from pLS-123-3′ UTR (containing predicted target seed region 5′GCAATT3′) and matched control pLS-123-3′ UTRmut (seed changed to 5′CGTACA3′) in the presence of miRNA mimics. Data are presented as the Log_2_ ratios of luciferase activities of targeting to non-targeting miRNA mimics (i.e. MR5057-miR-3p to negative control). The data shown were compiled from three experiments, values represent means between experiments and error bars represent standard deviations; *indicates p < 0.05.

CyHV-3 *ORF123* is an early gene [82] that encodes a putative deoxyuridine triphosphatase (dUTPase) [81]. In order to determine if it was a potential target for MR5057-miR-3p as predicted, we first investigated whether MR5057-miR-3p could regulate CyHV-3 dUTPase levels. As antibodies are not yet available for this protein, a vector was generated from which a recombinant N-terminally epitope-tagged dUTPase (rdUTPase) was expressed from *ORF123* linked to its own 3′ UTR. This vector was then used to co-transfect HEK293 cells in conjunction with either a MR5057-miR-3p mimic or negative control miRNA mimic and rdUTPase levels were subsequently determined by Western blotting. It can be seen that rdUTPase levels were greatly reduced in the presence of MR5057-miR-3p mimic relative to when negative control mimic was used (Fig 12A). In order to confirm a role for the predicted MR5057-miR-3p target site in the *ORF123* 3′ UTR, the latter was inserted immediately downstream of the luciferase coding sequence in the 3′ UTR reporter vector pLightSwitch-3′ UTR. A matching plasmid was also generated in which bases complementary to the seed region of MR5057-miR-3p were eliminated from the target site by mutagenesis. These vectors were then separately used to co-transfect HEK293 cells in conjunction with either a MR5057-miR-3p mimic or negative control miRNA mimic. Ablation of the 3′ UTR target seed region led to a significant recovery of luciferase activity in the presence of MR5057-miR-3p when compared to the negative control mimic (p < 0.05) (Fig 12B). This indicated that the predicted target site was important for MR5057-miR-3p interaction with the 3′ UTR of *ORF123*. Together these results are evidence that MR5057-miR-3p interacts with the 3′ UTR of *ORF123* and can downregulate the level of CyHV-3 dUTPase.

## Discussion

Following *de novo* prediction of pre-miRNAs on the CyHV-3 genome, we used filters that were based on the attributes of known viral pre-miRNAs to distinguish high quality predictions from background noise. Interestingly, notable differences between genomes known to encode pre-miRNAs and those predicted not to do so, only became apparent following filtering. Although it is highly likely that the majority of those remaining predictions did not represent genuine pre-miRNAs, this strategy nonetheless proved to be of use in identifying viral genomes with pre-miRNA coding capacity. Several studies suggest that many pre-miRNAs arise through the random formation and continuous mutation of hairpin structures on genomes [6,83–85]. Thus, it stands to reason that viral genomes that have evolved to form greater numbers of random hairpins that are structurally consistent with Drosha/Dicer substrates might be more likely to encode pre-miRNAs. And indeed, the prediction statistics for the CyHV-3 genome suggested that it may have evolved in a way that was more conducive to generating miRNAs, relative to other viral genomes. Interestingly, four high-probability CyHV-3 pre-miRNAs identified in this study were also included in the top 25 CyHV-3 pre-miRNA VMir predictions (Dataset F in S1 File), indicating the effectiveness of our noise reduction strategy and the VMir scoring algorithm.

The proportions of small RNA reads that were of viral origin were quite low in both infections, in line with observations with other viruses [86,87] however this is likely to vary with experimental conditions [87]. Considering that all 156 CyHV-3 ORFs are transcribed during lytic infections [82], it is unsurprising that the majority of unique CyHV-3 transcripts sequenced represented mRNA degradation products. In comparison, fewer unique CyHV-3 reads mapped to non-coding regions, but these were much more abundant in terms of read count. The majority of these represented MR5057-miR-3p and its associated isomiRs which accounted for 37% and 82% of total CyHV-3 reads in the H361 and N076 infections respectively, however it is not unusual for one or a few viral miRNAs to dominate an entire viral ‘miRNAome’ [88,89]. Here, as in other studies, dominant small RNAs that mapped adjacent to pre-miRNA terminal loops were scored as miRNAs, although some of these were not necessarily the dominant isomiR at their loci in both infections. Despite this, the 5′ ends had a strong tendency to remain unchanged among the dominant isomiRs, which may reflect a need to keep seed regions intact. This has been observed elsewhere among miRNAs from both higher organisms [19,90] and viruses [88,91–93] and may be regulated in a tissue-specific manner *in vivo* [74,94].

Notably, none of the proposed mature miRNA duplexes (Fig 2) display features that are consistent with Drosha/Dicer processing (i.e. typical 2 nt 3′ overhangs at both ends). This is most likely because the miRNAs highlighted are in fact derived from different mature miRNA duplexes (i.e. different isomiRs). There may be several reasons for this: (i) methodological bias could result in preferential representation of specific isomiRs in the deep sequencing data [95–99], (ii) differences in 5′ base composition may result in different patterns of RISC incorporation, or (iii) a combination of both. Current evidence suggests that that Dicer cleaves with a high degree of precision [100] and as Dicer cleavage sites are determined by the overhangs created by Drosha [100,101], isomiR ends may be set by alternative Drosha processing of pre-miRNA ends. Furthermore, because 3′ end modification of miRNAs occurs after initial Dicer/Drosha cleavage [100], all isomiRs derived from the same mature miRNA duplex should at least still retain a common 5′ end. We therefore reasoned that if the miRNA/miRNA* pairs in Table 2 were derived from separate mature miRNA duplexes, the appropriate reads corresponding to these original duplexes may be present among specific isomiR subsets that map to the opposite arm, specifically those that share a common 5′ end that resulted in a 2 nt 3′ overhang when duplexed with miRNAs/miRNAs* in question. Within such isomiR subsets, the isomiR that resulted in 2 nt 3′ overhangs on both ends of the proposed duplex was taken to represent the original partner strand in the absence of any 3′ end modification. Indeed, for nine out of the twelve miRNA/miRNAs* in Table 2, such reads from the opposite arm were identified (Dataset Y in S1 File), thus providing the supporting evidence to indicate that they were derived from RNase III cleavage events.

All high-probability CyHV-3 pre-miRNAs had alignment signatures that were distinctly “miRNA-like”. However the signatures for MD1111 and MR5075 displayed significant isomiR/isomoR overlap along with additional transcripts occurring adjacent to moRNAs. Similar instances have nonetheless been observed with *bone-fide* pre-miRNAs elsewhere [73,102]. Although consistently different from random RNA degradation fragments, the signatures for MD1111 and MR5075 do not fit neatly into the model of miRNA biogenesis. They may in fact be “transitional” pre-miRNAs [72,76,85], representing evolutionarily nascent Drosha/Dicer substrates that are not as robustly processed. Rather than displaying such “relaxed” or lower confidence alignment signatures, other transitional pre-miRNAs may be just inefficiently processed [83,85], which may account for other high-probability/putative CyHV-3 pre-miRNAs such as MD9812, MD11410, MR6201 and MD11704 which had low miRNA read counts. Despite this, the miRNA sequences derived from these six possible transitional pre-miRNAs were highly consistent between both infections, with 8/12 remaining unchanged at either end and 10/12 unchanged at the 5′ end. Analysis of the isomiRs from CyHV-3 miRNAs showed that there was significant bias towards 3′ end heterogeneity, as is usually the case with isomiRs from both higher organisms [74–76] and viruses [77,88,91,93,103–105]. Although 5′ processing was very consistent within most isomiR sets, some did not show much difference between the two ends in this respect and a small number displayed significant amounts of 5′ heterogeneity. While the latter is more unusual, similar patterns have been observed elsewhere [72,74,88].

Automated approaches to miRNA identification typically generate high numbers of false positives, however independent identification of the same pre-miRNA candidates by multiple methods has been shown to support the annotation of genuine pre-miRNAs [106]. Despite the fact that most candidates identified by miRDeep2 and MIREAP occurred in protein-coding regions only one of these was independently identified by both programs. The notable lack of independent identification among such candidates reflects the fact that subtle miRNA-like patterns identified in ORFs are more likely to be the result of sporadic background noise from random mRNA degradation. Conversely, such interference may also mask genuine miRNA-like alignment signatures or read enrichment in these regions. Some viral pre-miRNAs do indeed occur within ORFs and 3′ UTRs [87,107], however it is unclear if these were identified due to a lack of (or in spite of) such interference from degraded mRNAs, the latter being more of an issue during lytic infections with higher levels of viral transcription and increased RNA turnover [108].

The additional discovery of CyHV-3-encoded moRNAs is interesting. This recently described class of small RNAs have also been observed in several other herpesviruses [91,103,104,109–111]. It is apparent from the CyHV-3 pre-miRNA alignment signatures that the 5′ or 3′ ends of the moRNAs are largely ‘fixed’, corresponding to the 5′ or 3′ ends of adjacent CyHV-3 miRNAs (or major isomiRs). This is consistent with previous observations and speculation that both classes of small RNAs may arise from a common pre-miRNA cleavage event [22,72,73,103]. MoRNA abundance is not linked to miRNA/miRNA* patterns and instead 5′ arm moRNAs are generally more dominant [111,112], although MR5075-moR-5p is the only CyHV-3 moRNA to display such 5′ arm dominance. The functions of these non-coding RNAs remain unclear and unlike miRNAs, evidence suggests that they accumulate in the nucleus [112], although they may also be inefficiently incorporated into the RISC [111].

DNA microarray hybridization strongly supported the annotation of all miRNAs derived from MR5057 and MD11776 as being genuine. The most highly expressed miRNA was MR5057-miR-3p, and although efficiently processed by Dicer, the pre-miRNA nonetheless accumulated in sufficiently high levels to also be detectable by Northern blotting. This may be due to (i) high levels of pri-miRNA expression, (ii) highly efficient Drosha processing of the pre-miRNA or (iii) both. As the low VMir score for MR5057 suggested that its structure deviated from that of typical pre-miRNAs, it is possible that Dicer processing of this pre-miRNA may not be efficient and that the high levels of processed MR5057-miR-3p may instead be just a consequence of high levels of its pre-miRNA. It is also possible that MR5057 and MR5075 share the same pri-miRNA, given their extremely close proximity to one another. If this is the case, the difference in the relative levels of these pre-miRNAs/miRNAs implies that they must be processed at very different rates, similar to EBV miRNAs from the BART cluster [113].

Similar relative CyHV-3 miRNA expression levels were consistently observed in non-synchronous *in vitro* infections. Although the latter may not be representative of the situation *in vivo* where other factors including tissue type and host immune response are important, the relative levels of MR5057-miR-3p and MD11776-miR-3p were also maintained during infection *in vivo*. It is interesting to note that most herpesvirus miRNAs identified during lytic infections remain expressed during latency, albeit with different expression levels [28,88,105,107,114–116]. If also expressed during latency, CyHV-3 miRNAs could therefore have potential as better diagnostic targets than viral DNA which can be difficult to reliably detect at this stage of infection [44,45].

MiRNA genes are generally not conserved between different viral species. Instances of conservation or high sequence similarity have only been observed between closely related viruses [63,77,109,117–119] but this is not always the case [119–121]. Here, we show CyHV-3 pre-miRNA sequence similarities with CyHV-1 and CyHV-2 that are highest in the regions encoding the seed regions of MD11776-miR-3p and MD11704-miR-5p, suggesting the presence of functionally important miRNAs at these loci in all three viruses. We note that owing to the position of genomic DNA insertions, the MR5075-miR-5p miRNA identified in this study does not exist in the CyHV-3 TUMST1 MR5075 pre-miRNA, clearly indicating that this miRNA is not essential in this strain.

Functional miRNA target sites are generally located in the 3′ UTRs of mRNAs however they can also be located, albeit less frequently, within protein coding regions and 5′ UTRs [65,122–126]. The fact that MR5057-miR-3p occurs antisense to the CyHV-3 *ORF43* (an early gene of unknown function [127]) makes the latter an obvious potential target. However, target sites in ORFs are generally not as functionally active as those within 3′ UTRs, most likely due to interference from translation [26,65,125,128]. In addition to simple seed pairing, there are many other subtle characteristics associated with *bone-fide* miRNA target sites, including local sequence composition, seed base pairing stability, pairing outside the seed and target site accessibility. Taking these criteria into account, we identified and validated the 3′ UTR of the viral dUTPase-encoding *ORF123* as a target of MR5057-miR-3p. This interaction may potentially contribute to a dynamic process that controls CyHV-3 genome replication/gene expression during various stages of viral infection. In addition to their important role in nucleic acid metabolism, viral dUTPases have also been shown to function as signalling molecules that modulate cellular responses including the anti-viral immune response [129]. Furthermore, as *ORF123* and its 3′ UTR occur within the 3′ UTR of the upstream gene *ORF122* (an early gene of unknown function [81]) (Fig 11B), MR5057-miR-3p may serve to simultaneously regulate the expression of both. CyHV-3 miRNAs may also target host mRNAs and this can be assessed once the *C*. *Carpio* genome has been sufficiently annotated. Some CyHV-3 miRNAs may be functional orthologues of host miRNAs, notably the MD11776-miR-5p seed is identical to the host miRNA ccr-miR-29a whose human orthologue has been shown to be involved in regulating apoptosis [130]. Such similarities to host miRNA seed regions may allow the virus to tap into host miRNA regulatory networks.

In conclusion, our findings confirm that CyHV-3 expresses at least two pre-miRNAs during lytic infections, namely MR5057 and MD11776. Further experimental scrutinisation of the four high-probability and two putative CyHV-3 pre-miRNAs identified in this study, may support their classification as CyHV-3 encoded pre-miRNAs. In accordance with the miRBase nomenclature system, the official names proposed for the CyHV-3 pre-miRNAs MR5057 and MD11776 are CyHV3-mir-1 and CyHV3-mir-2, respectively, in order of their genomic location. In accordance with the same system, the official names proposed for the remaining high-probability and putative candidates are CyHV3-mir-3 (MD1111), CyHV3-mir-4 (MR6201), CyHV3-mir-5 (MR5075), CyHV3-mir-6 (MD9812), CyHV3-mir-7 (MD11410) and CyHV3-mir-8 (MD11704). Further studies on the role and *in vivo* expression of CyHV-3 miRNAs and moRNAs are likely to add significantly to our understanding of the biology of this economically important virus and will determine if these small RNA sequences have the potential to serve as biomarkers for the identification of latently infected fish. In addition, the identification of CyHV-3 pre-miRNA homologues opens up other lines of inquiry into the existence of miRNAs encoded by other members of the *Alloherpesviridae*.

## Supporting Information

S1 Fig Qualitative and quantitative coverage plots of small RNA deep sequencing reads mapping to the CyHV-3 genome (SeqMap output) . Reads from CyHV-3 H361 and N076 isolates are displayed on upper and lower data tracks respectively. ORFs and polyA signals are also shown along the bottom of the figure. Features on the forward strand are indicated in red and those on the reverse strand in blue.
(PDF)

S1 File Combined file. Dataset A, Details of adaptors and primers used for small RNA deep sequencing library preparation. Dataset B, VMir analysis of six other herpesvirus genomes known to encode pre-miRNAs. The names of all known pre-miRNAs on these viral genomes (miRBase Release 19) are listed. Details of VMir predictions corresponding to these known pre-miRNAs are also displayed. In addition two other viral genomes that are unlikely to encode pre-miRNAs were also analysed in the same manner. Abbreviations: EBV-Epstein-Barr virus, HSV-1-Herpes Simplex-1, HCMV—Human Cytomegalovirus, KSHV—Kaposi’s sarcoma-associated Herpesvirus, MDV—Marek’s Disease virus, MGHV68—Mouse Gammaherpesvirus 68. HHV-3—Human herpesvirus type 3 (also known as Varicella Zoster virus), HHV-7—Human herpesvirus 7. Dataset C, Summary of VMir pre-miRNA prediction statistics from six herpesvirus genomes known to encode pre-miRNAs (listed in Dataset B). The number of known pre-miRNAs from each virus was based on information available from miRBase (Release 19). These are also compared to the VMir pre-miRNA prediction statistics generated from the CyHV-3 genome. Abbreviations: EBV—Epstein-Barr virus, HSV-1—Herpes Simplex-1, HCMV-Human Cytomegalovirus, KSHV—Kaposi’s sarcoma-associated Herpesvirus, MDV—Marek’s Disease virus, MGHV68—Mouse Gammaherpesvirus 68. Dataset D, Summary of VMir pre-miRNA prediction statistics from two herpesvirus genomes that have not been shown to encode pre-miRNAs. Abbreviations: HHV-3-Human herpesvirus type 3 (also known as Varicella Zoster virus), HHV-7-Human herpesvirus 7. Dataset E, Characteristics of VMir predicted MHPs corresponding to known viral pre-miRNAs listed in Dataset B. This data was used to establish relevant minimum cut-off values for Score and Relative Window Count when eliminating the least likely pre-miRNA candidates predicted in VMir analysis of the CyHV-3 genome. Dataset F, VMir predicted CyHV-3 pre-miRNAs (MHP details listed) outside protein coding regions post filter. Absolute-WC refers to the WC of the MHP, Relative-WC refers to the combined WC of the MHP and all SHPs (only MHPs are listed for each prediction). Dataset G, 21 High and low probability pre-miRNA candidates identified on the CyHV-3 genome using the non-automated approach. Dataset H, Sequences representing putative miRNAs and moRNAs mapping to 15 low probability pre-miRNA candidates identified using the non-automated approach. Dataset I, Pre-miRNA classifier analysis of both low and high probability CyHV-3 pre-miRNA candidates identified using the non-automated approach. Dataset J, Alignment signatures (from both infections) of small RNAs mapping to 21 CyHV-3 pre-miRNA candidates identified using the non-automated approach. Dataset K, Enrichment quantification of reads from 21 CyHV-3 putative pre-miRNAs identified using the non-automated approach. The quantitative information displayed represents a Log_2_ ratio of the observed base density in the region divided by the overall base density on the same data track. Loci that are enriched in terms of read counts relative to their surrounding genomic regions appear as positive values (i.e. above the axis) in the quantitative information tracks. Features (reads/read-stacks, ORFs and PolyA signals) on the forward strand are shown in red and those on the reverse strand are shown in blue. Reads mapping to forward and reverse strands are kept on separate data tracks. Relevant PolyA signals are indicated with circles. The black arrows indicate the directionality of all pre-miRNAs and mRNAs. Reads mapping to the low probability pre-miRNA loci in do not collectively form discrete stacks and are not enriched in terms of read counts relative to flanking genomic regions. Conversely, reads mapping to high probability pre-miRNA loci do form discrete stacks and are all enriched in terms of read counts relative to flanking genomic regions. In addition unlike the low probability pre-miRNA loci, which are located in intergenic regions, the high probability pre-miRNA loci are all located in 3′ UTRs immediately downstream of ORFs. Dataset L, CyHV-3 pre-miRNA candidates identified by miRDeep2 in the H361 and N076 infections. Pre-miRNA candidates with miRDeep2 scores that were <0 and repeated pre-miRNA candidates are not included in this list. More detailed information and alignment signatures for the pre-miRNA candidates shown here are given in Dataset N. Dataset M, CyHV-3 pre-miRNA candidates identified by MIREAP in the H361 and N076 infections. Repeated pre-miRNA candidates and pre-miRNA candidates that lack miRNA* reads in both infections are not included in this list. More detailed information and alignment signatures for the pre-miRNA candidates shown here are given in Dataset O. Dataset N, miRDeep2 output containing additional information and alignment signatures for all CyHV-3 pre-miRNA candidates identified in the two infections. Dataset O, MIREAP output containing additional information and alignment signatures for all CyHV-3 pre-miRNA candidates identified in the two infections. Dataset P, Summary of results from automated identification of miRNAs from sequencing data. Dataset Q, Offset values for both ends of individual isomiRs (defined as reads that had ≥60% of their bases overlapping with the miRNA) from all thigh probability pre-miRNAs in Table 3. The start and end positions for each individual isomiR, were used to calculate the offset values relative to start and end positions of the miRNAs (highlighted in bold). IsomiRs representing <0.1% of the combined miRNA and isomiR read count (for the miRNA in question) were eliminated from further analysis. In order to avoid the risk of including partially degraded isomiRs which could possibly skew the results, transcripts <19nt in length were also eliminated from further analysis as per Riley KJ-L, Rabinowitz GS, Steitz JA. Comprehensive analysis of Rhesus lymphocryptovirus microRNA expression. J Virol. 2010;84: 5148–5157. doi:10.1128/JVI.00110-10. Dataset R, Analysis of isomiR end heterogeneity for putative miRNAs from high probability CyHV-3 pre-miRNAs. MiRNAs with 3′ End: 5′ End ratios that are <1 are highlighted in bold. Dataset S, Supporting Data from microarray hybridization. (Top) Hybridization signals and MOB values for endogenous positive controls and spike in controls for all samples. (Middle) Hybridization signals from probes targeting miRNAs from CyHV-3 pre-miRNA candidates MR5057 and MD11776 and associated control probes. (Bottom) DNA microarray analysis of 21 high and low probability pre-miRNA candidates identified using the non-automated method. The MOB values for each pre-miRNA represent that of the miRNA probe (5′ or 3′ arm) with the highest corrected signal. MOB (Multiples of Background) values that exceeded the cut-off value for a given hybridization are highlighted in bold. Dataset T, Details of samples used for *in vivo* testing. Names and geographic co-ordinates of locations for fish collections. Ct values from Stem Loop RT-PCR assays targeting MR5057-miR-3p and MD11776-miR-3p are also displayed. Corresponding values for ccr-let-7a expression ranged from 21.57–29.19. Dataset U, BLASTN alignments of CyHV-3 pre-miRNA sequences to the CyHV-1 and CyHV-2 genomes. These results relate to the CyHV-1 and CyHV-2 sequences underlined in Fig 10. Dataset V, Alignment of the CyHV-3 pre-miRNA MD11776 to VMir predicted CyHV-2 pre-miRNA MD4320 (genomic co-ordinates 149470–149574). This predicted CyHV-2 pre-miRNA corresponds to the potential homologue identified through BLASTN alignments between MD11776 and the CyHV-2 genome (see Fig 10 and underlined sequence). Nucleotides that are conserved in both CyHV-3 and CyHV-2 are in red. This MHP has a VMir score of 203.1 and a relative-WC of 39 which are both consistent with the VMir prediction characteristics of known viral pre-miRNAs (Dataset E). There is predicted to be only one shorter variant (i.e. Sub-Hairpin or SHP) of this MHP, which folds within 22 analysis windows (i.e. absolute-WC value of 22) making it more stable within the local sequence context than the MHP itself which has an absolute-WC value of 17. Furthermore, unlike any other potential CyHV-3 pre-miRNA homologue sequences, this SHP was classified as a real pre-miRNA by both MiPred (Confidence: 81.5%) and CSHMM (Likelihood score: 0.23102204942883192). Dataset W, CyHV-3 miRNA seed matches with known viral miRNAs. Seed regions (2–7) are marked in bold. Dataset X, CyHV-3 miRNA seed region matches to known host miRNAs. Seed regions (2–7) are marked in bold. Dataset Y, Proposed mature miRNA duplex structures for all putative CyHV-3 miRNAs. Individual miRNA/miRNA* reads are identifiable by the name assigned in Table 2. These duplex structures were supported by the identification of additional reads from opposite arms of their respective pre-miRNAs that form duplexes with the correct 2 nt 3′ end overhangs. In cases where the corresponding strand from the mature miRNA duplex was not detected, the proposed hypothetical duplexes are still displayed and the corresponding strands were recorded as “not detected’.
(XLSX)
