# Supplementary material for: Identification and Characterization of Cyprinid Herpesvirus-3 (CyHV-3) Encoded MicroRNAs
Source: PLoS One. 2015 Apr 30;10(4):e0125434. doi: 10.1371/journal.pone.0125434 (PMC4416013; doi:10.1371/journal.pone.0125434)
Supplement: S1 Fig — . Reads from CyHV-3 H361 and N076 isolates are displayed on upper and lower data tracks respectively. ORFs and polyA signals are also shown along the bottom of the figure. Features on the forward strand are indicated in red and those on the reverse strand in blue. (PDF) [file pone.0125434.s001.pdf]

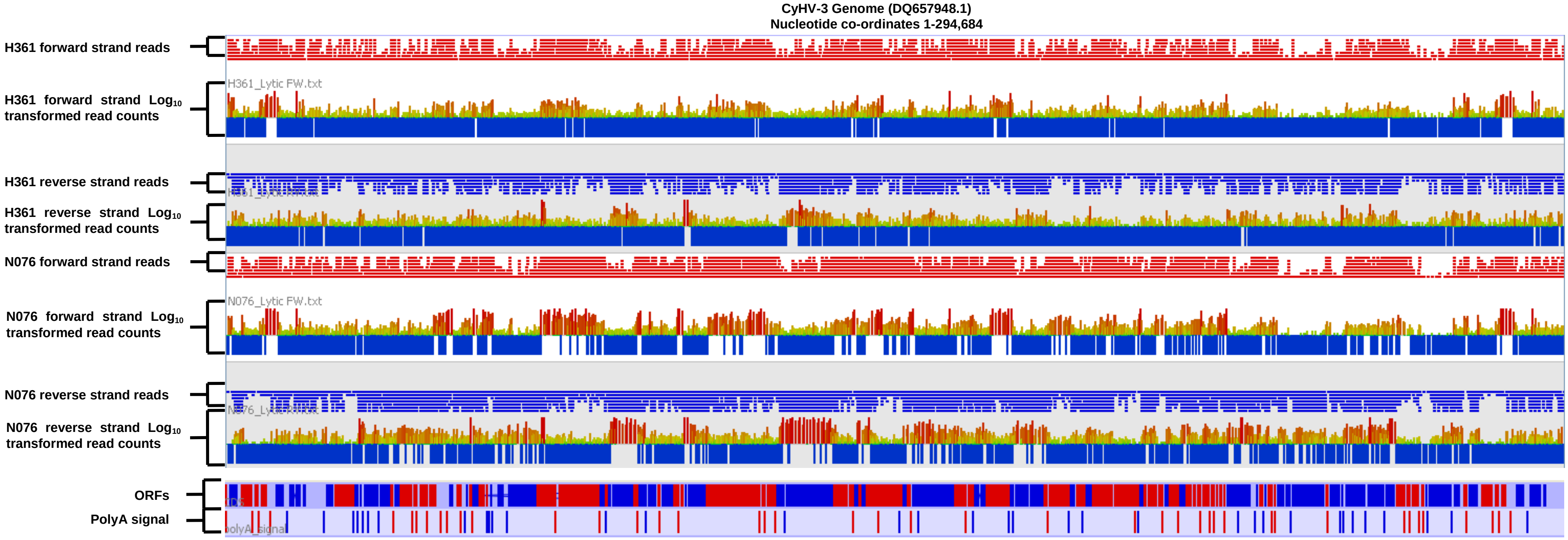

S1 Fig. Qualitative and quantitative coverage plots of small RNA deep sequencing reads mapping to the CyHV-3 genome (SeqMap output). Reads from CyHV-3 H361 and N076 isolates are displayed on upper and lower data tracks respectively. ORFs and polyA signals are also shown along the bottom of the figure. Features on the forward strand are indicated in red and those on the reverse strand in blue
